# Novel nonperipheral octa-3-hydroxypropylthio substituted metallo-phthalocyanines: synthesis, characterization, and investigation of their electrochemical, photochemical and computational properties

**DOI:** 10.3906/kim-2008-48

**Published:** 2021-02-17

**Authors:** Nilgün KABAY, Yasemin BAYGU, Metin AK, İzzet KARA, EsraNur KAYA, Mahmut DURMUŞ, Yaşar GÖK

**Affiliations:** 1 Department of Biomedical Engineering, Pamukkale University, Denizli Turkey; 2 Tavas Vocational School of Higher Education, Pamukkale University, Denizli Turkey; 3 Department of Chemistry, Pamukkale University, Denizli Turkey; 4 Department of Physical Education, Pamukkale University, Denizli Turkey; 5 Department of Chemistry, Gebze Technical University, Kocaeli Turkey; 6 Department of Chemical Engineering, Uşak University, Uşak Turkey

**Keywords:** Metallo-phthalocyanines, cyclic voltammetry, computational chemistry, photodynamic therapy, photochemical properties

## Abstract

The current study describes the synthesis, electrochemical, computational, and photochemical properties of octa (3-hydroxypropylthio) substituted cobalt (II) ( **4** ), copper (II) ( **5** ), nickel (II) ( **6** ) and zinc(II) ( **7** ) phthalocyanine derivatives. These novel compounds were characterized by elemental analysis,^1^H,^13^C NMR, FT-IR, UV-Vis, and MS. The redox behaviors of these metallo-phthalocyanines were investigated by the cyclic voltammetric method. The optimized molecular structure and gauge-including atomic orbital (GIAO)^1^H and^13^C NMR chemical shift values of these phthalocyanines in the ground state had been calculated by using B3LYP/6–31G(d,p) basis set. The outcomes of the optimized molecular structure were given and compared with the experimental NMR values. The photochemical properties including photodegradation and singlet oxygen generation of zinc(II) phthalocyanine were studied in DMSO solution for the determination of its photosensitizer behaviors.

## 1. Introduction

Phthalocyanines (Pcs) and their metal complexes have been studied for a long time, and they are still the matters of intense investigation. They show various exceptional properties and they havepotential applications in different scientific and innovative areas like nonlinear optics [1], electrochromic imaging systems [2], chemical detectors [3–5], solar cells [6], photovoltaic optics, molecular electronics [7], liquid crystals [8], semiconductors [9], laser dyes [10], optical storage devices [11], catalyst [12] and photodynamic therapy (PDT) [13]. The developing utilization of phthalocyanines as cutting edge materials in the recent decade and they have empowered the blend of new materials which vary as far as the central metal ion and peripheral substituents [14].

Electrochemical properties of phthalocyanines in the electrolytic solution, are dependent ontheir energy values of the HOMOs and LUMOs of the frontier orbitals [15]. Electrochemical properties of the proposed compounds may have the possible potential usage in electrocatalysis, electrosensing, and electrochromic devices. Electron donating alkylthio substituted phthalocyanines are also inherently electron-rich p-type semiconductors [16,17].

The numerous applications of zinc(II) phthalocyanines in the field of medicine, molecular electronics, magnetic devices, chemical sensors depend on their photophysical and photochemical properties. The photochemical properties of these compounds, especially, singlet oxygen quantum yield and photostability were also investigated for photodynamic therapy applications [18]. Therefore, they are widely used in cancer treatment as novel generation photosensitizers. Photosensitizers are desirable to have a long wavelength. In this manner, they have an effective curing performance over deep skin cancer types. However, due to having low energy, photosensitizers decrease the possible harmful effect of light irradiation. [19,20]. Phthalocyanines were known as second-generation photosensitizers in PDT of cancer. They have long-wavelength absorption and highly effective singlet oxygen generation abilities. For this reason, they are suitable for use in cancer treatment [21].

In this study, the novel metallo-phthalocyanines wherein the 3-hydroxypropylthio groups connected to nonperipheral positions of the Pc macrocycle were synthesized. Electron donating sulfur groups are known to shift the Q-band to the long wavelength in nonperipheral positions that is desirable for potential PDT applications. The newly synthesized compounds have been characterized by^1^H,^13^C NMR, UV-Vis, FT-IR, micrOTOF mass, electrochemical and computational studies as well as elemental analysis. The photochemical properties such as singlet oxygen generation and photodegradation of zinc(II) phthalocyanine was also investigated to determine possible usage of this compound as a photosensitizer for cancer treatment by photodynamic therapy technique. The theoretical^1^H and^13^C NMR data of the optimized geometry were also compared with the experimental chemical shift values.

## 2. Experimental

All information about the used materials, equipment, synthesis, electrochemical measurements singlet oxygen and photodegradation quantum yields as photophysical properties and theoretical calculations were showed in the “Supplementary materials”.

## 3. Results and discussion

### 3.1. Synthesis and characterization

**Scheme 1:**
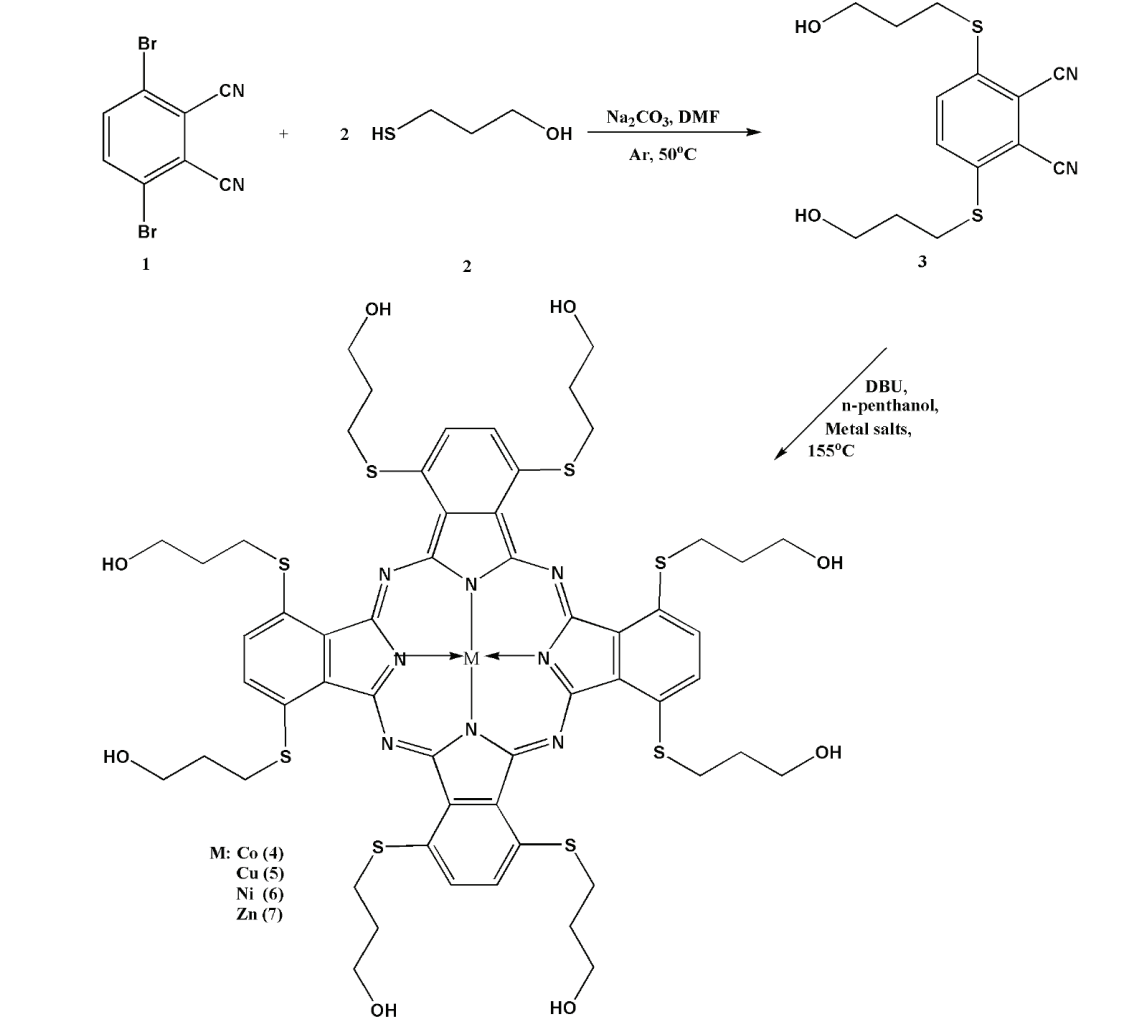
The synthesis route of the phthalonitrile and metallophthalocyanines.

3,6-bis(3-hydroxypropylthio)phthalonitrile (3) was synthesized via a condensation reaction of 3,6-dibromophthalonitrile [22] with 3-mercapto propanol under very convenient conditions with a better yield than the first synthesis result (38%) (Scheme 1) [23]. This compound was prepared previously by the SNAr reaction of 3,6-(4’-methylphenyl-sulfanyloxy) phthalonitrile with 3-mercapto-propanole. In the^1^H NMR spectrum of this compound, resonances at δ = 3.49, 1.73, 3.17, and 4.67 ppm should be related to OCH2, CH2, SCH2, and –OH protons, respectively. The aromatic protons appeared as a dublet at δ = 7.81–7.78 ppm as expected (Figure S1).^13^C NMR spectrum of 3 showed the presence of characteristic carbon resonances of C≡N groups at δ = 116.95 ppm, that can be attributed to the formation of 3,6-disubstituted phthalonitrile. The other chemical shifts at δ = 132.9, 141.2, 145.3, 59.6, 31.9, and 29.7 ppm could be related CN-ArC, ArC, S-ArC, OCH_2_, SCH_2_, and CH_2_moieties, respectively (Figure S2). These NMR signals (Table 1) are in accordance with the published results [23]. In the FT-IR spectrum of this molecule showed the characteristic vibrations for the C≡N groups at 2220 cm^-1^(Figure S3).

**Table 1:** ^1^H and^13^C chemical shifts of compound 3 (experimental and theoretical values).

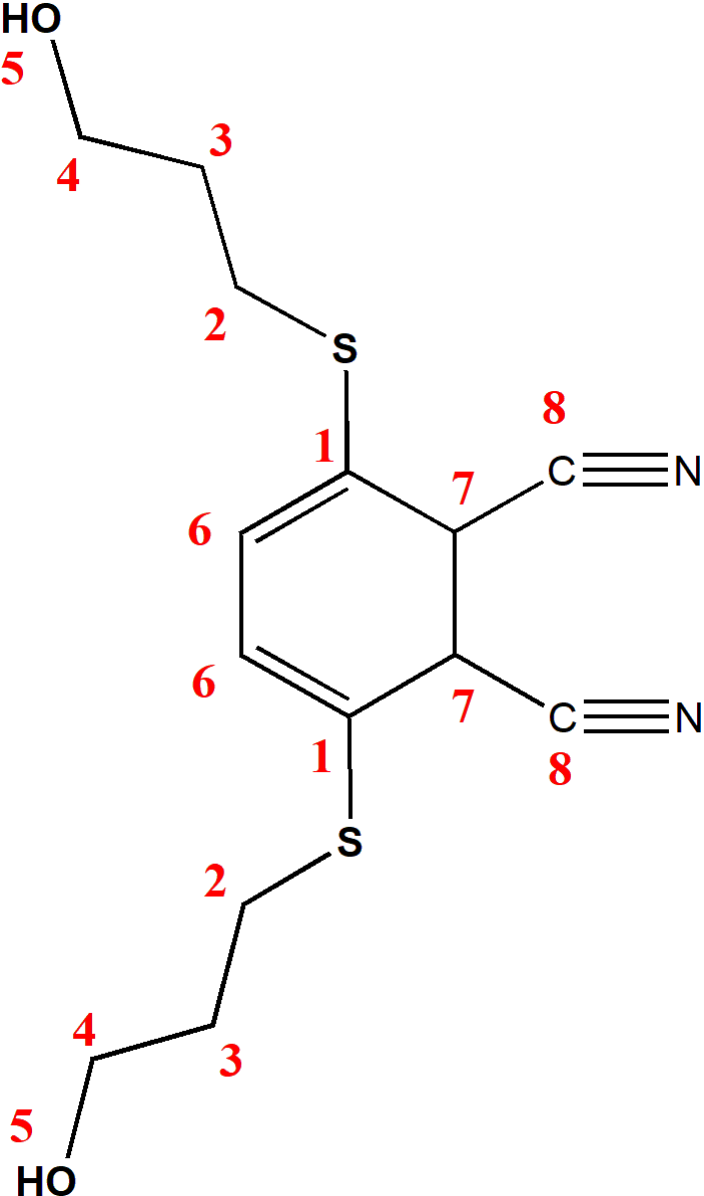	Atoms	Exp.	Gas phase	DMSO
	C1	145.27	141.99	143.74
	C2	31.87	32.06	32.31
	C3	29.74	32.13	32.39
	C4	59.55	62.29	61.59
	C6	132.99	119.57	123.10
	C7	141.15	113.72	111.04
	C8	116.05	105.13	108.02
	H2	3.17	2.08	3.08
	H3	1.73	2.74	1.98
	H4	3.49	4.01	4.02
	H5	4.67	0.19	0.80
	H6	7.81	7.11	7.63

Metallo-phthalocyanines ( **MPc** ) **(4** –7) were synthesized by the reaction of 3 with anhydrous metal salts (CoCl_2_, NiCl_2_, CuCl_2_, and Zn(OAc)_2_) in n-pentanol in the presence of catalytic amounts of DBU under an argon atmosphere (Scheme 1). The shared features of all new products were performed by spectroscopic methods and elemental analysis such as UV-Vis, FT-IR,^1^H NMR (for compounds 3, 6, and 7),^13^C NMR (for compounds 3 and 7) and MS (micrOTOF).

In the^1^H NMR spectra of nickel(II) (6) and zinc(II) (7) phthalocyanines in DMSO-d6, the characteristic resonances of aromatic protons were observed at δ = 7.74–7.58 or 7.98 ppm, respectively. The other signals of compound 6 and 7 due to hydroxypropyl groups as multiplets at δ = 1.95 (6) or singlet at 2.11 ppm (7) for -CH2- protons, broad chemical shift at δ = 3.66 (6) and dublet at 3.77 ppm (7) for OCH2 protons, broad singlet at δ = 3.33 (6) ppm as superimposed H2O proton, and singlet δ = 3.45 (7) ppm for SCH2 protons and broad peaks at δ = 4.70 (6), 4.75 (7) ppm concerning OH (Figures S4 and S5).^13^C NMR spectra concerning C≡N signals at δ = 116.9 ppm belonging to precursor compound (3) disappeared in the case of NiPc and ZnPc formations. In addition to that, the appearance of novel signals at δ = 145.5 ppm and δ = 152.7 related to the inner core of phthalocyanines also indicated the formulation of metallo-phthalocyanine structures (Figures S6 and S7). The other^13^C NMR data of these molecules were almost identical to those of the precursor molecule (3) as anticipated. In the MS spectra of NiPc and ZnPc measured by the micrOTOF technique proved proposed structure due to the molecular ion peaks which observed at m/z = 1291.9 [M]+ and 1298.9 [M]+, respectively (Figures S8 and S9). In the FT-IR spectra of the compounds (4–7) (Figures S10–S13), the stretching vibrations concerning C≡N groups at 2221 cm^-1^belong to phthalonitrile (3) disappear after the cyclotetramerization reaction. The deformation of these vibrations confirmed the formation of phthalocyanines. The rest of the FT-IR spectra showed very close similarity to the starting compound. The mass spectra of compounds 4 and 5 recorded by micrOTOF technique also confirmed the molecular ion peaks at m/z = 1291.0 [M+H]+ and 1297.2 [M]+, respectively (Figures S14 and S15).

**Figure 2:**
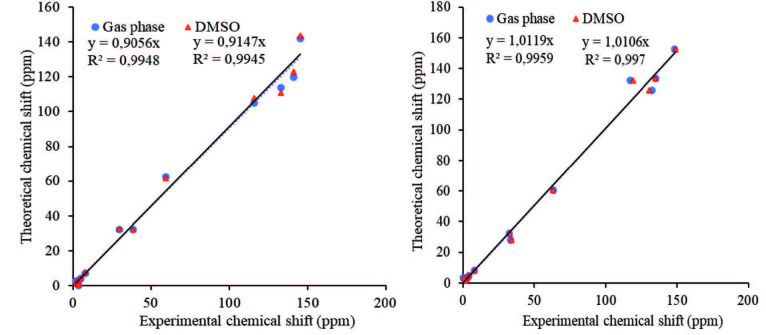
The correlation graphs between the experimental and theoretical ^1^H and ^13^C chemical shift values of the molecules in DMSO.

Theoretical^1^H and^13^C NMR chemical shifts of the compound 3 and ZnPc were calculated from B3LYP/6–31G (d,p) (see in the optimized molecular structure of the ZnPc in the ground state, Figure 1). The calculated NMR resonances concerning phthalonitrile compound 3 and ZnPc were given in Tables 1 and 2, respectively. The optimized geometric parameters of the ZnPc compound (bond lengths, bond angles, and dihedral angles) by B3LYP methods with 6–31G(d,p) as the basis set were presented in Tables S1 and S2. The correlations (Figure 2) between the experimental and calculation of the chemical shift values of the compounds are described by the equations of dcal (ppm) = 0,953 dexp + 0,6972 (R2 = 0,983) for compound 3 (Table 1) and dcal(ppm) = 0,9738 dexp + 0,6852 (R2 = 0,9915) for ZnPc (Table 2), respectively.

**Figure:**
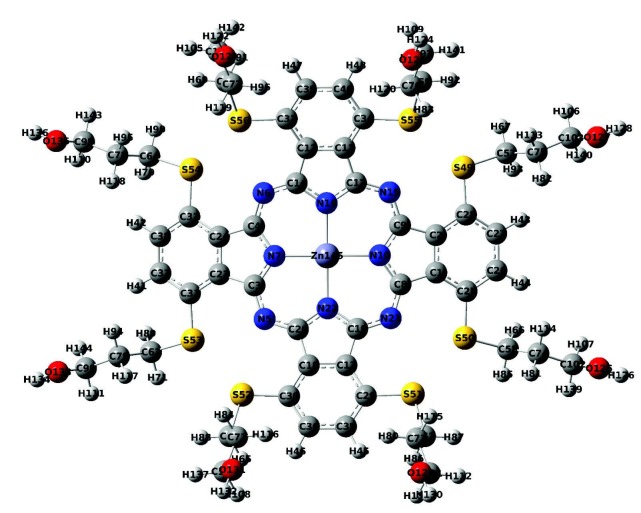
Optimized geometry of the ZnPc in the ground state.

**Table 2:** ^1^H and^13^C chemical shift of ZnPc (experimental and theoretical values).

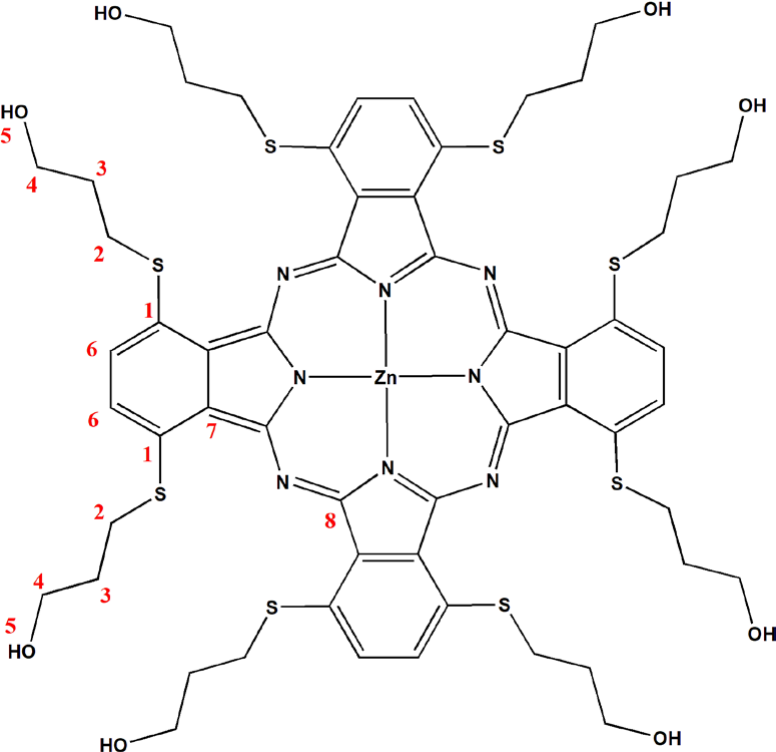	Atoms	Exp.	Gas phase	DMSO
	C1	133.66	135.07	134.94
	C2	32.26	32.36	32.78
	C3	28.23	33.48	34.10
	C4	60.41	63.14	62.96
	C6	125.57	117.07	119.32
	C7	132.69	132.46	130.59
	C8	152.68	148.46	149.21
	H2	3.45	3.07	3.19
	H3	2.11	2.46	2.19
	H4	3.77	4.07	4.08
	H5	4.75	0.19	0.75
	H6	7.98	7.79	7.99

### 3.2. Ground state electronic absorption spectra

**Figure 3:**
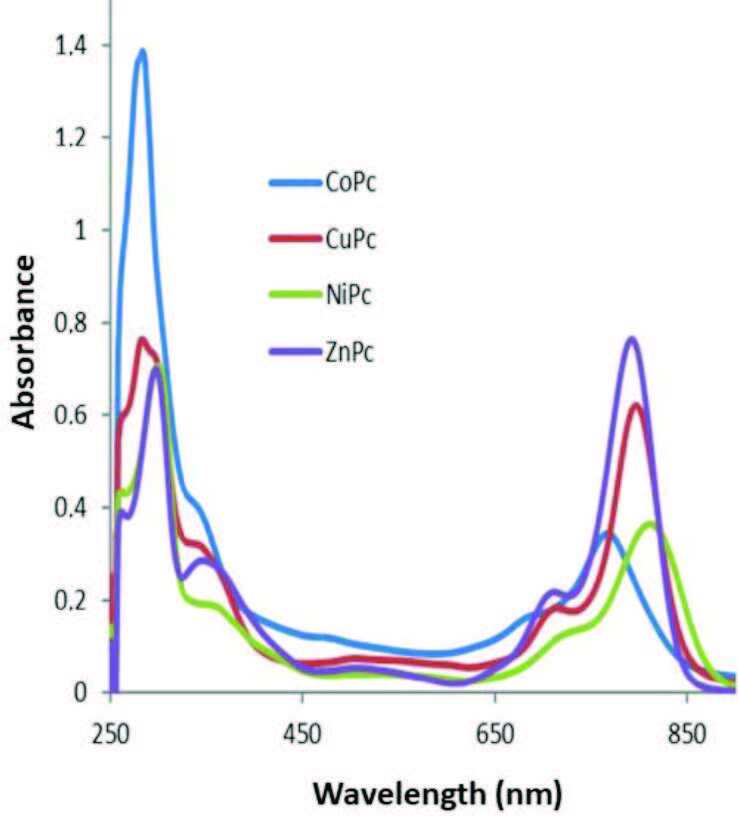
The UV-Vis spectra of MPc (1 × 10^-5^ M in DMSO).

Phthalocyanine compounds show two strong absorption bands in their electronic absorption spectroscopy that correlate to π → π* transitions. One of them is the so-called Q-band and seen at around 600–800 nm, and the other is called as B band and arise approximately 300–450 nm [24]. The ground-state electronic absorption spectra of Ni(II), Co(II), Cu(II) and Zn(II) phthalocyanines were measured in DMSO (Figure 3). The most of phthalocyanines show characteristic absorption band in the visible region approximately at around 600–750 nm named the Q-band and in the UV region at around 300–400 nm named B or Soret band [25]. The Q-band absorption in DMSO of the four metallated phthalocyanine can be aligned in the sequence Ni(II) > Cu(II) > Zn(II) > Co(II). The Q-bands were seen at 811, 797, 792, and 767 nm for compounds 6, 5, 7, and 4, respectively. These single absorptions in lower energy regions should be related π→π* transitions of the phthalocyanine cores. The Q-bands of compounds are significantly red-shifted among the metallo-phthalocyanines. It is well known that the electron-releasing groups such as alkylthio are bound to eight α-benzo positions of the phthalocyanine skeleton, the Q-band absorptions shift to longer wavelength. The transition metal ions have been settled in the phthalocyanine core may be expected they greatly affect absorption properties. Ni(II), Cu(II) and Co(II) have a similar electronegativity [26], so that the effect of electronegativity on the red-shifted is inferred to be similar [27]. The absorption maxima of 4 and 7 shifts to the shorter wavelength in the order of Co(II) and Zn(II) as central metals in the phthalocyanine core [28]. Ni(II) phthalocyanines have maximum red as an unusual shift among the metallatedphthalocyanines can be attributed the perfect planarity of the d8 electronic configuration of this metal [14].

**Figure 4:**
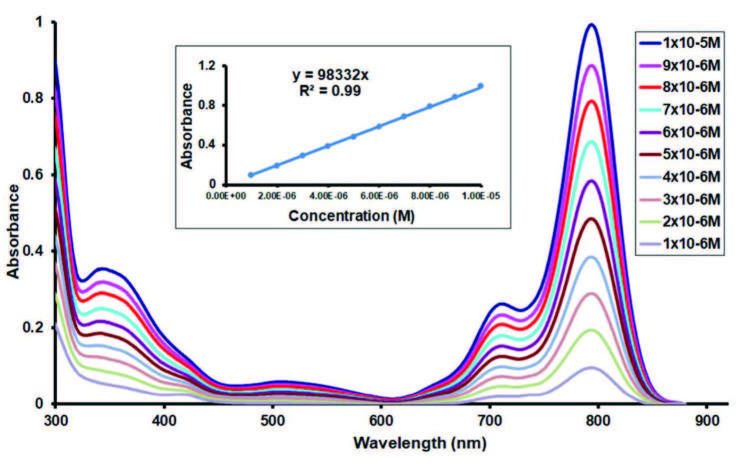
Electronic absorption spectral changes for complex 7 in DMSO at different concentrations (Inset: Plot of absorbance versus concentration).

Aggregation is usually portrayed as a coplanar association of rings proceeding from monomer to dimer and higher order complexes. There are lots of parameters for aggregation in phthalocyanines; concentration, the nature of the solvent, nature of the substituents, complexed metal ions, and temperature [29]. The Q-band absorption maximum was independent of concentration and followed the Beer–Lambert law with a constant extinction coefficient in the studied concentration range [Figure 4 as an example for **ZnPc** (7)] for all studied metallo-phthalocyanines and these phthalocyanines did not exhibit any aggregation in the studied concentration range.

### 3.3 Electrochemical studies

**Figure 5:**
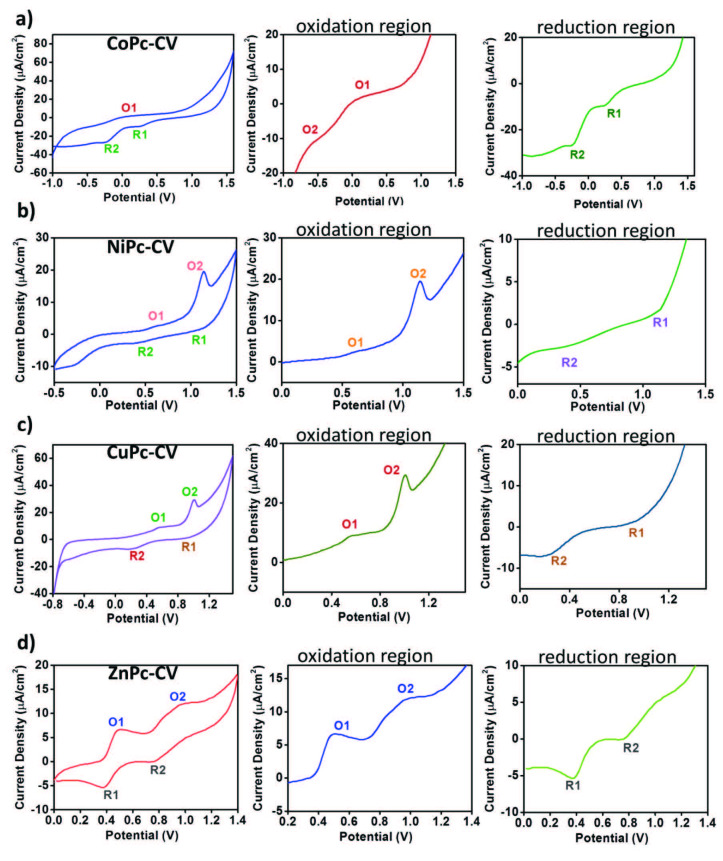
CV responses of synthesized metallo-phthalocyanines a) CoPc, b) NiPc, c) CuPc, d) ZnPc recorded in the cathodic and anodic potential side in DCM:DMF (0.8:0.2)/ TBP6 electrolyte system on an ITO working electrode.

Voltammetric analyses of metallo-phthalocyanines have been performed with cyclic voltammetry (CV) as mentioned above. Figure 5 demonstrates the CV responses of the synthesized metallo-phthalocyanines recorded in the cathodic and anodic potential side in DCM:DMF (0.8:0.2)/TBP6 electrolyte system on an ITO working electrode.

**CoPc** gives nonquasi-reversible metal-based reduction at 0.25 V (R1). Also, it is thought that nonquasi-reversible Pc based oxidation and reduction reactions were observed at 0.1 V(O1) and –0.25 V(R2). Most of the studies in the literature on reduction properties of the MPc complexes including that such complexes have two reduction processes as one metal- and one ring-based [30,31].

Cyclic voltammetry graph of **NiPc** showed two oxidation peaks at 1.14 and 0.62 V and consecutive reduction peaks at 1.00 V and 0.38 V. When the electrochemical behavior of **CuPc** is examined, oxidation and reduction peak values have found to be lower than those of **NiPc** . In terms of **CuPc** , these values are 1.0 V and 0.56 V for oxidation and 0.18 V and 0.88 V for reduction. **ZnPc** had the lowest oxidation and reduction peak potential values among those of the other studied phthalocyanine derivatives. Compared to **NiPc** which has the oxidation peak values at 1.14 V and 0.62 V, **ZnPc** had lower redox peak values at 0.98 V and 0.49V. This result may be due to the smaller atomic radius of Zn metal comparing to the other metals’ atomic radii.

**Figure 6:**
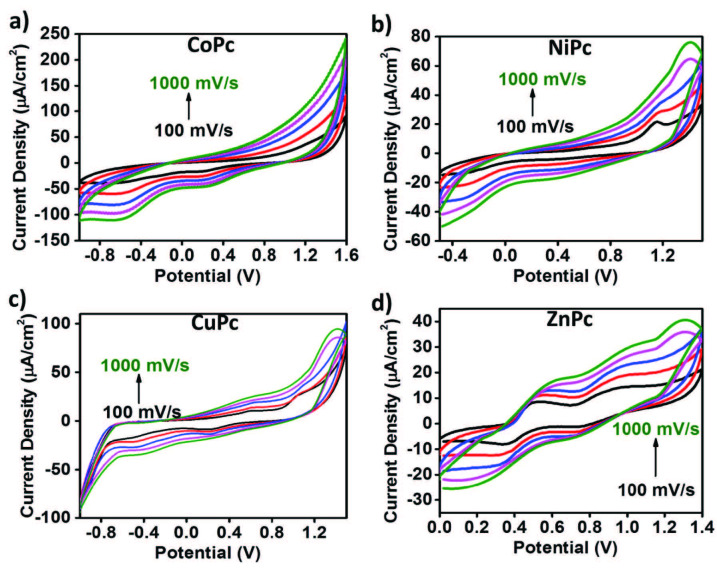
CV responses of synthesized metallo-phthalocyanines at different scan rates a) CoPc, b) NiPc, c) CuPc, d) ZnPc recorded in DCM:DMF (0.8:0.2)/TBP6 electrolyte system.

The speed of the applied potential can be changed by changing the scan rate in cyclic voltammetry experiments. Higher peak current values were obtained at high scan rates due to a reduction in the size of the diffusion layer [32]. Figure 6 shows a series of cyclic voltammograms recorded at different scan rates for an electrolyte solution containing metallo-phthalocyanine. The linearity of the peak current values with the square root of the scan rates proved that the electrochemical reaction on the electrode surfaces was diffusion controlled as expected [32].

### 3.4. HOMO-LUMO studies

The electrostatic potential map of a molecule supply knowledge about the electron acceptor and electron donor regions. This knowledge may help us to see the relationships between the atoms regarding intramolecular and intermolecular hydrogen bonds. The distinctive values of the electrostatic potential at the area of the map are referred to by varied colours: blue refers to the most positive electrostatic potential, red refers to the most electronegative electrostatic potential site and green refers to the zero potential sites.

The electronic properties of a molecule can be calculated depending on HOMO and LUMO energies. In the calculations, the electron affinity ( *A* =–LUMO) and the ionization potential ( *I* =–HOMO) are the basic parameters. The other parameters such as absolute electronegativity (c=( *I+A* )/2), softness ( *S* =( *I-A* )/2), and absolute hardness (h=( *I-A* )/2) can be calculated accordingly.

**Figure 7:**
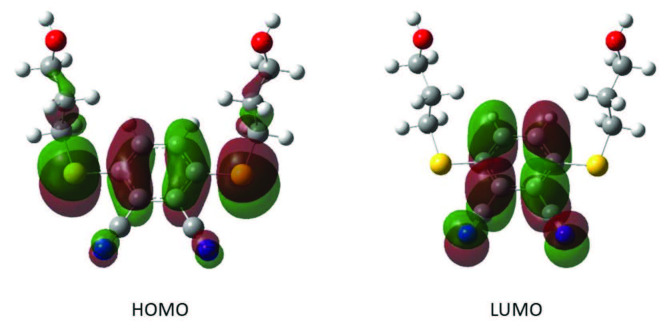
The HOMO and LUMO energies of the compound 3 with B3LYP/6–31G(d,p) basis set in gas phase.

**Figure 8:**
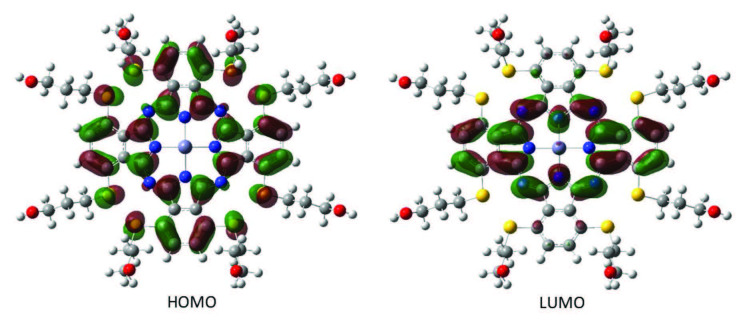
The HOMO-LUMO energies of the ZnPc.

The dispersions of the HOMO and LUMO orbitals calculated for the B3LYP/6–31G(d, p) level for the compounds 3 and ZnPc were shown in Figures 7 and 8, respectively. In our calculations, ZnPc had a total of 1588 orbitals out of which 339 were filled and the rest were 1249 empty orbitals. The orbital numbered as 339 accounted for HOMO and 340 accounted for LUMO orbitals. The corresponding energy values were calculated as –4.29 eV for the HOMO and –2.42 eV for the LUMO energies with B3LYP/6–31G(d, p) level. The parameters for the 3,6-bis-(3-hydroxypropylthio)phthalonitrile were calculated at the same levels and the results were presented in Table 3.

**Table 3:** Electronic properties for the phthalonitrile (3) and ZnPc compounds.

	Phthalonitrile (3)			ZnPc		
Electronic parameters	6–31g(d)	6–31g(d,p)	6–31g+(d,p)	6–31g(d)	6–31g(d,p)	6–31g+(d,p)
eV	4.3009	4.2996	4.1644	1.6878	1.6857	1.6504
l(Å)	288.28	288.36	297.72	734.6	735.51	751.25
Oscillator strengths	0.1665	0.1458	0.2951	0.4347	0.4356	0.4374
HOMO (au)	–0.22186	–0.22195	–0.23012	–0.15748	–0.15774	–0.16678
LUMO (au)	–0.07989	–0.08017	–0.09137	–0.08906	–0.08939	–0.09940
ΔE=LUMO-HOMO	3.86	3.86	3.78	1.86	1.86	1.83
TD/LUMO-HOMO	4.30	4.30	4.16	1.69	1.69	1.65
I (eV)	6.04	6.04	6.26	4.29	4.29	4.54
A (eV)	2.17	2.18	2.49	2.42	2.43	2.70
χ (eV)	4.11	4.11	4.37	3.35	3.36	3.62
Hardness(η)	1.93	1.93	1.89	0.93	0.93	0.92
Softness(s)	0.52	0.52	0.53	1.07	1.08	1.09
µ = -(I + A)/2 = - χ	–1.93	–1.93	–1.89	–0.93	–0.93	–0.92
ω = µ2 / (2η)	0.966	0.965	0.944	0.465	0.465	0.458
Dipole moment (debye)	12.084518	12.036222	12.379576	6.017804	5.869903	7.233204
Polarizability (α) (a.u)	211.008667	212.453118	237.359667	724.789333	729.525000	792.483000
Hyperpolarizability (β) (a.u)	214.054379	215.011406	574.413256	387.135619	394.940022	1518.758510

**Figure 9:**
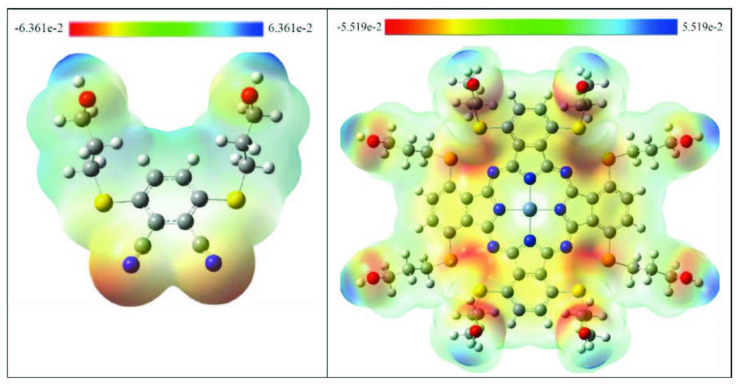
The MAP surface obtained at B3LYP/6–31G(d,p) level for phthalonitrile and ZnPc compounds.

As shown in Figure 9, the red region was localized on the nitrogen atoms and vicinity of the sulphur atoms in both the phthalonitrile and the **ZnPc** , whereas the blue region was delocalized on the OH groups. Hence, it was found that the **ZnPc** was useful to both bond metallically, and it has intermolecularly interacted. This result also supports the evidence of the charge analyses part.

NBO analysis is a tool for the determination of intramolecular interactions. The NBO analysis is used to specify the interactions between filled and empty orbitals of a molecule with the help of DFT method [33–35]. The NBO analysis, especially charge transfer, indicates the role of intermolecular orbital interaction in the compound. In tandem with this, the stabilization energy E(2) linked with electron delocalization between donor and acceptor is predicted for each donor NBO (i) and acceptor NBO (j) as follows:

where qi is the orbital occupancy of the ith donor, Ej and Ei are the diagonal elements (orbital energies) and F(i,j) is the off-diagonal NBO Fock matrix element. The hyper conjugative σ→σ* interactions play an extremely significant role in the molecule represent the weak departures from a strictly localized natural Lewis structure that constitutes the primary “noncovalent” effects [36]. The results of the NBO analysis of the **ZnPc** collected with B3LYP/6–31G(d,p) basis set presented in Table S3.

The interactions between C25-C26 (π*) and π*(C1-C2), C27-C28 (π*) and π*(C1-C2), the stabilization of 275.24 kcal/mol, which denotes larger delocalization. According to Table S3, C25-C26 is rich in electrons since close to the electron release group. That is why it is a donor. In contrast, C1-C2 is acceptor because the electron is poor. The interaction between the C8-N10 (σ *) π*(C19-N21), C3-N5 (π*) π*(C23-C31), C4-N7 (π*) π*(N6-C14), C13-N16 (π*) π*(C9-N15), C19-N21 (π*) π*(C17-C29), C20-N22(π*) π*(C3-N5) also represent the larger delocalization. The E(2) value is essential chemically and may be exploited as a measure of the intramolecular delocalization.

The calculated visible absorption maxima at TD–B3LYP/6–31G(d,p) of λ which are a function of the electron availability were displayed in Table 3. The most likely transition for the molecule is the HOMO-LUMO transition at 339→340 because the maximum f = 0.4356 (oscillator strength) value is in the excited state-1 at 735.51 nm. HOMO-LUMO+1 transition was calculated at 339→341 molecular orbital, excited state-2: 735.21 nm. Typically, the energy bandgap in inorganic materials is ~ 1.5 eV, in organic materials is in the range of 1.5–3.5 eV. In accordance with this, the compound is capable of being a potential molecule for inorganic semiconductor materials [37]. Additionally, according to ligand, this value reveals that the compound becomes more conductive electrically.

### 3.5. Singlet oxygen generation properties

**Figure 10:**
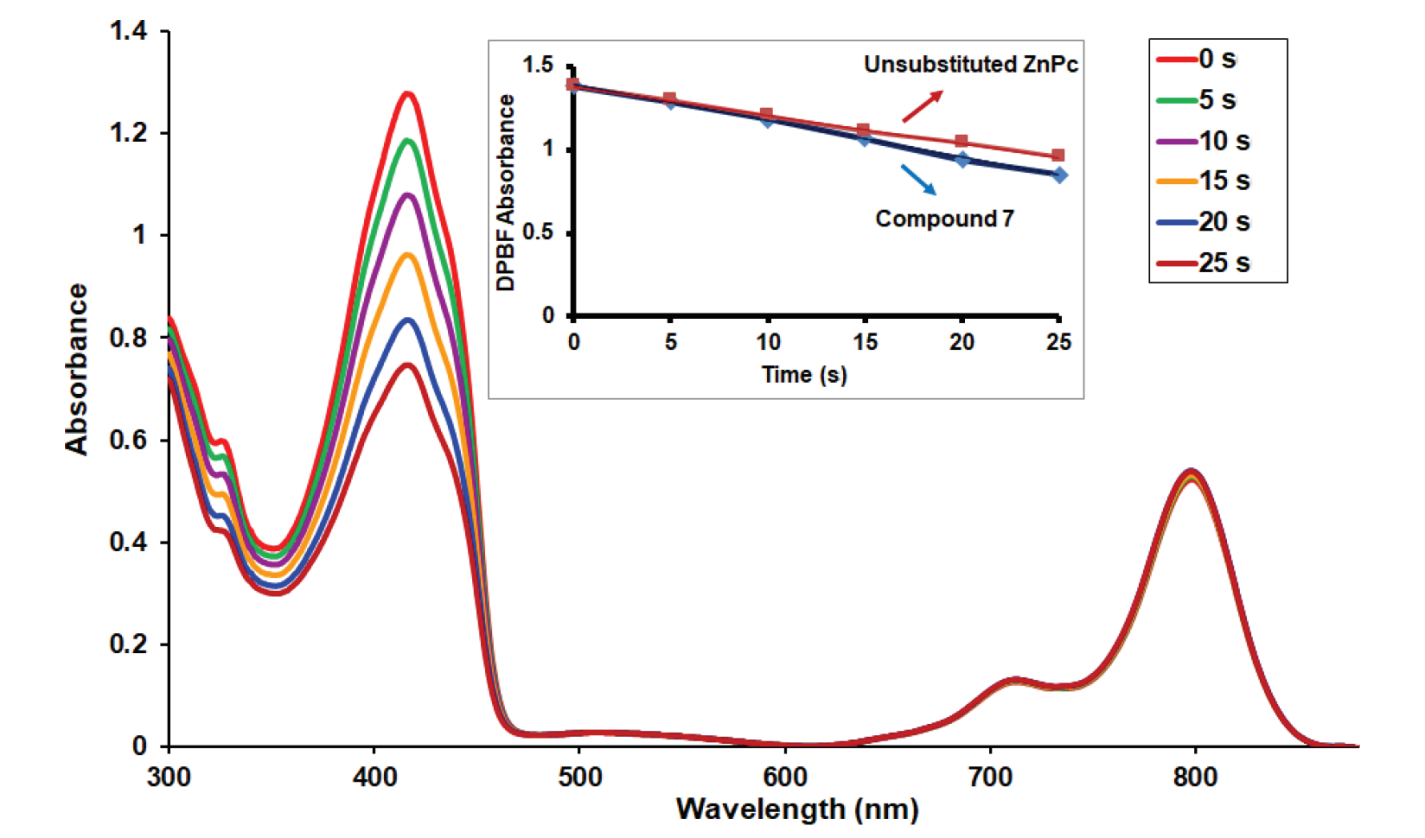
Electronic absorption spectral changes during singlet oxygen determination. This determination was for compound 7 in DMSO at a concentration of 1.0 × 10^-5^ M using DPBF at a concentration of 1.0 × 10^-4^ M (Inset: Plot of DPBF absorbance versus time).

PDT is a treatment for cancer where light, molecular oxygen, and photosensitizer are used in combination to produce cytotoxic forms of oxygen such as singlet oxygen. The generation of singlet oxygen is the key to show PDT potential of the compounds [38]. The singlet oxygen production of studied zinc(II) phthalocyanine (7) was determined with the chemical method in DMSO. 1,3-diphenylisobenzofuran (DPBF) was used as a singlet oxygen scavenger which causes the formation of endoperoxide species. A time-dependent decrease of DPBF absorbance at 417 nm was observed for phthalocyanine photosensitizer 7. There was no change in the Q-band intensity during the ΦΔdeterminations and it supports that studied phthalocyanine did not show any degradation by used light irradiation (Figure 10). The singlet oxygen production of studied zinc(II) phthalocyanine (7) was found higher compared to unsubstituted zinc(II) phthalocyanine in DMSO (Figure 10, inset). The singlet oxygen generation properties of other metallo-phthalocyanines (4, 5, and 6) studied in this work did not investigate due to paramagnetic behavior of the used Co, Ni, and Cu metal ions the cavities of these phthalocyanines because paramagnetic metal ions reduce the photoactivity of the molecules.

The synthesis of the phthalocyanine compounds bearing different alkylthio groups on the phthalocyanine ring were given in the literature but the photochemical properties of these derivatives were studied limitedly. On the other hand, the phthalocyanine derivatives substituted at the nonperipheral octa positions of the phthalocyanine macrocycle are very rare in the literature. The studied zinc(II) phthalocyanine (7) showed higher singlet oxygen production in comparison with other octa nonperipheral substituted photosensitizers containing different alkylthio groups such as 2-propoxy, benzyloxy or 3,5 bis(benzyloxy)benzyloxy groups [39]. Similarly, the studied zinc(II) phthalocyanine (7) showed higher singlet oxygen production in comparison with alkylthio substituted pyridoporphyrazines [40]. Additionally, the phthalocyanine 7 exhibited similar singlet oxygen generation when compared to the nonperipherally octa-sulfanyl substituted zinc phthalocyanine [41].

### 3.6. Photodegradation studies

**Figure 11:**
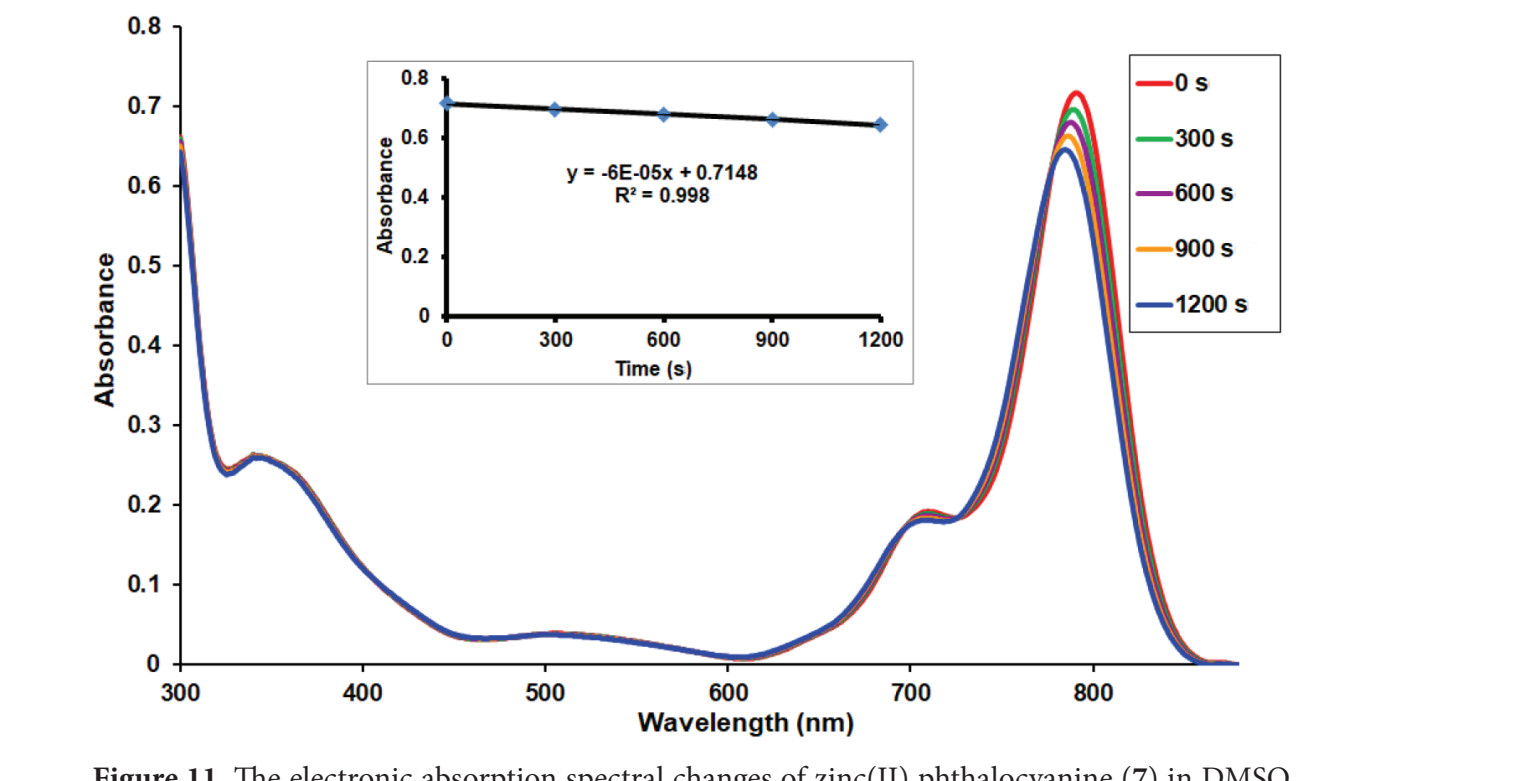
The electronic absorption spectral changes of zinc(II) phthalocyanine (7) in DMSO under light irradiation revealing the vanishing of the Q-band at 5 min intervals (Inset: Plot of absorbance vs. time).

Photodegradation quantum yield can be used to study the stability of photosensitizer during the photocatalytic reaction in PDT [42]. The current study shows that photodegradation properties of studied zinc(II) phthalocyanine (7) were determined in DMSO by monitoring the collapse of their absorption bands underused light irradiation with increasing time (Figure 11). The Φd value of zinc(II) phthalocyanine was found the order of 3.79 × 10–5 (between 10–3 and 10–6 for ideal photosensitizer) in DMSO [21]. The Φd value of the investigated zinc(II) phthalocyanine (7) was found slightly higher than unsubstituted zinc(II) phthalocyanine (Φd = 2.61 × 10–5) [18]. On the other hand, the phthalocyanine 7 exhibited lower photodegredation quantum yield value when compared to the non-peripherally octa-sulfanyl substituted zinc phthalocyanine [41] which means that the studied zinc(II) phthalocyanine (7) exhibited higher stability to light irradiation.

## 4. Conclusion

In this study, the phthalonitrile derivative substituted with 3-hydroxypropylthio groups at 3 and 6 positions as ligand and its non-peripheral octa substituted metallo-phthalociyanines [M =Zn(II), Ni(II), Cu(II) and Co(II)] were synthesized and characterized. Electrochemical properties of the proposed compounds also investigated because of these kinds of compounds show the possible potential usage in electro-catalysis, electrosensing, and electrochromic devices. When the ΔE values ​​in Table 3 are compared, the **ZnPc** compound is electrically more conductive than phthalonitrile. In this electrical conductivity, zinc(II) plays an important role. Besides, the molecular geometry and GIAO^1^H and^13^C NMR chemical shift values of the molecule in the ground state had been estimated by applying B3LYP with 6–31G(d,p) basis set. Also, the photochemical properties such as singlet oxygen generation and photodegradation under light irradiations were studied for the determination of possible photosensitizer ability of zinc(II) phthalocyanine derivative 7. These properties of the other studied metallo-phthalocyanines did not investigate because of the paramagnetic behavior of metal ions (Co, Ni, and Cu) in the cavities of these phthalocyanines. The absorbance of the new zinc(II) phthalocyanine (7) was studied in DMSO solutions at different concentrations for determination of the most suitable concentration for further photochemical properties. The singlet oxygen production of this phthalocyanine (7) was determined in DMSO using a chemical method. The singlet oxygen production of studied zinc(II) phthalocyanine (7) was found higher compared to unsubstituted zinc(II) phthalocyanine in DMSO. In this study, the photodegradation behavior of the zinc(II) phthalocyanine (7) was determined in DMSO. The Φd value of zinc(II) phthalocyanine was found in the order of 3.79 × 10–5 in DMSO. This value is slightly higher than unsubstituted zinc(II) phthalocyanine [37,43].

Supplementary Materials
