# Supplementary material for: Novel nonperipheral octa-3-hydroxypropylthio substituted metallo-phthalocyanines: synthesis, characterization, and investigation of their electrochemical, photochemical and computational properties
Source: Turk J Chem. 2021 Feb 17;45(1):143–56. doi: 10.3906/kim-2008-48 (PMC7925307; doi:10.3906/kim-2008-48)
Supplement: Supplementary file 1 — Supplementary Materials [file turkjchem-45-143-sup001.pdf]

Supplementary materials

1. Materials and methods

1.1. Experimental

All reagents were supplied from commercial suppliers. All reactions were carried out under the argon atmosphere. All solvents were dried and purified according to standard methods [S1]. ^{1}H NMR and ^{13}C NMR spectra were measured by a Varian Mercury 300 NMR spectrometer. FT-IR and mass spectra were recorded on a Perkin-Elmer Spectrum One FT-IR spectrometer and on a micrOTOF mass spectrometer. UV-Vis spectral measurements were performed by Shimadzu UV-1601 spectrometer at room temperature. Melting points were measured by an electrothermal apparatus and are uncorrected. 3,6-dibromo phthalonitrile was synthesized according to the literature procedures [S2].

2. Synthesis

2.1. 3,6-bis-(3-hydroxypropylthio)phthalonitrile (3)

A mixture of 3,6-dibromo phthalonitrile (**1**) (1.43 g, 5 mmol), excess amount of anhydrous Na_2CO_3 (2.65 g, 25 mmol) and 3-mercapto-1-propanol (**2**) (1.15 g, 12.5 mmol) in dry DMF (25 mL) were placed in a round-bottom two flask under argon atmosphere. This suspension was stirred at 50 °C for 10 h. The reaction mixture was monitored by TLC [silica gel (chloroform:methanol)(95:5)]. At the end of this period, the reaction mixture was cooled to room temperature and filtered. The filtrate was evaporated under reduced pressure to dryness and then the solid product purified by column chromatography on silica gel using the mixture of chloroform:methanol (95:5) as eluent to give a pale yellow solid. Yield: 0.58 g (38%); m.p. 143 °C (140 °C in reference [S3]). FT-IR (ν, cm^{-1}): 3236 (O-H), 3067 (Ar-H), 2929–2848 CH_2), 2220 (C≡N), 1527, 1471, 1434, 1284, 1143, 1035, 822; ^{1}H NMR (300 MHz, DMSO-d_6): δ 7.81–7.78 (d, 2H, Ar-H), 3.49 (m, 4H, OCH_2), 4.67 (s, 2H, OH), 3.17 (m, 4H, S-CH_2), 1.73 (m, 4H, CH_2). ^{13}C NMR (75 MHz, DMSO-d_6): δ 145.3, 141.2, 132.9, 116.9, 59.6, 31.9, 29.7. Anal. calcd. for $C_{14}H_{16}N_2S_2O_2$: C, 54.52; H, 5.23; N, 9.08. Found: C, 54.33; H, 5.40; N, 9.23.

2.2. 1,4,8,11,15,18,22,25-octakis(3-hydroxypropylthio)phthalocyaninato metal complexes (4–7)

A mixture of 0.5 mmol (0.154 g) 3,6-bis-(3-hydroxypropylthio)phthalonitrile and 0.182 mmol anhydrous metal salt (23.7 mg cobalt chloride, 23.7 mg nickel chloride, 24.5 mg copper (II) chloride or 33.3 mg zinc acetate) in 3 mL of *n*-pentanol and 3 drops 1,8-diazabicyclo[5.4.0]undec-7-ene (DBU) was heated and stirred at 155 ˚C for 24 h under an argon atmosphere in a Schlenk tube. After this time, the mixture was chilled out to room temperature and the precipitate was filtered off and then washed subsequently with chloroform, water, and acetone. After that, the products were purified by Soxhlet extraction with chloroform and then dried in vacuo over dried $MgSO_4$.

2.2.1. Cobalt (II) phthalocyanine(4)

Yield: 0.105 g (65%); m.p. > 300 ˚C. FT-IR (**ν**, cm^{-1}): 3238 (-OH), 3057 (Ar-H), 2923–2868 (alkyl-CH), 1690 (C=N), 1567, 1434, 1281, 1153, 1041, 928; UV-Vis λ_{max} (nm) (log **ε**) in DMSO: 767 (4.54), 701 (4.24), 377 (4.33), 283 (4.59). MS: *m/z* 1291.03 $[M]^+$ (calculated MS: 1291.2) . Anal. calcd. for $C_{56}H_{64}N_8O_8S_8Co$; C, 52.03; H, 4.99; N, 8.67. Found: C, 51.86; H, 4.69; N, 8.40%.

2.2.2. Copper (II) phthalocyanine(5)

Yield: 0.102 g (63%); m.p. > 300 ˚C. FT-IR **ν** (cm^{-1}): 3290 (-OH), 3057 (Ar-H), 2923–2868 (alkyl-CH), 1644, 1585, 1427, 1280, 1154, 1036; UV-Vis λ_{max} (nm) (log **ε**) in DMSO: 797 (4.79), 713 (4.27), 503 (3.88), 349 (4.49), 283 (4.88). MS: *m/z* 1297.2 $[M+2]^+$, 1358.1 $[M+K+Na+H]^+$ (calculated MS: 1295.2) . Anal. calcd. for $C_{56}H_{64}N_8O_8S_8Cu$; C, 51.85; H, 4.97; N, 8.64; found: C, 51.34; H, 4.72; N, 8.36%.

2.2.3. Nickel (II) phthalocyanine(6)

Yield: 0.115 g (71%); m.p. > 300 ˚C. FT-IR **ν** (cm^{-1}): 3241 (-OH), 3048 (Ar-H), 2929– 2875 (alkyl-CH), 1690, 1567, 1434, 1281, 1153, 1041; ^{1}H NMR (300 MHz, DMSO-d_6): 7.74–7.58 (br s, 8H, ArH), 4.70 (br s, 8H, OH), 3.66 (m, 16H, OCH_2), 3.33 (m, 16H, SCH_2, with d-DMSO proton), 1.95 (m, 16H, CH_2CH_2). ^{13}C NMR (75 MHz, DMSO-d_6): 145.5, 133.1, 131.7, 124.7, 60.4, 32.0, 28.1. UV-Vis λ_{max} (nm) (log **ε**) in DMSO: 811 (4.56), 736 (4.14), 525 (3.62), 363 (4.27), 300 (4.85). MS: *m/z* 1291.9 $[M+2]^+$, 1309.6 $[M+H_2O]^+$ (calculated MS: 1290.2). Anal. calcd. for $C_{56}H_{64}N_8O_8S_8Ni$; C,52.04; H, 4.99; N, 8.67; found: C, 51.65; H, 4.74; N, 8.38%.

2.2.4. Zinc (II) phthalocyanine(7)

Yield: 0.125 g (77%); m.p. > 300 ˚C. FT-IR **ν** (cm^{-1}): 3254 (-OH), 3052 (Ar-H), 2924– 2868 (alkyl-CH), 1644, 1557, 1435, 1280, 1142, 1041, 919; ^{1}H NMR (300 MHz, DMSO-d_6): 7.98 (s, 8H, ArH), 4.75 (s, 8H, OH), 3.77–3.76 (d, 16H, OCH_2),

3.45 (br s, 16H, SCH_2), 2.11 (m, 16H, CH_2CH_2). ^{13}C NMR (75 MHz, DMSO-d_6): 152.7, 133.7, 132.7, 125.57, 60.4, 32.3, 28.2. UV-Vis λ_{max} (nm) (log ε) in DMSO: 792 (4.88), 711 (4.34), 503 (3.75), 346 (4.46), 296 (4.85). MS: *m/z* 1298.9 $[M+3]^+$ (calculated MS: 1296.2). Anal. calcd. for $C_{56}H_{64}N_8O_8S_8Zn$; C, 51.78; H, 4.97; N, 8.63; found: C, 52.24; H, 4.77; N, 8.52%.

2.3. Electrochemical measurements

All electrochemical measurements were actualized with Ivium potentiostat using the three-electrode system at room temperature. The Pt and Ag wires were used as counter and reference electrodes, respectively. Optically transparent indium tin oxide (ITO) coated glass slides from Delta Technologies (7 × 50 × 0.5 mm thickness and 8 – 12 ohm.sq^{-1}) were used as a working electrode. Electrochemical characterizations of the materials were carried out in DCM/DMF (0.8 / 0.2) solution containing 0.1 M tetrabutylammonium hexafluorophosphate (TBP_6). The cyclic voltammetry technique was applied for the electrochemical characterization of materials. The ITO-coated glass electrode, Ag wire, and Pt wire have been plunged into the electrochemical cell. Different voltage has been applied on the working electrode through the potentiodynamic method and has been controlled using the Ivium Compact stat. The Ag wire electrode was calibrated versus Ag/AgCl (3M KCl) electrode.

2.4. Photochemical studies

2.4.1. Singlet oxygen quantum yields

Singlet oxygen production determinations were carried out using the experimental set-up described in the literature [S4]. Typically, a 3 mL portion of the respectively substituted zinc(II) phthalocyanine (**7**) solution (concentration = 1 × 10^{-5} M) containing the singlet oxygen quencher was irradiated in the Q band region with the photo-irradiation set-up described in the reference [S4]. Singlet oxygen production was determined in the air using the relative method using unsubstituted ZnPc as a standard. 1,3-diphenylisobenzofuran (DPBF) was used as a chemical quencher for singlet oxygen in DMSO. To avoid chain reactions induced by DPBFin the presence of singlet oxygen [S5],the concentration of quenchers (DPBF) was lowered to ~ 3 × 10^{-5} M. Solutions of sensitizer (1 × 10^{-5} M) containing DPBF was prepared in the dark and irradiated in the Q-band region using the setup described and degradation of DPBF at 417 nm was monitored.

2.4.2. Photodegradation quantum yields

Photodegradation quantum yield (Φ_d) determinations were carried out using the experimental set-up described in the literature [S4]. Photodegradation quantum yields were determined using Equation (1),

$$\Phi_d = \frac{(C_0 - C_t) . V . N_A}{I_{abs} . S . t} \quad (1)$$

where C_0 and C_t are the sample (**7**) concentration before and after irradiation respectively, V is the reaction volume, N_A is the Avogadro's constant, S is the irradiated cell area, it is the irradiation time and I_{abs} is the overlap integral of the radiation source light intensity and the absorption of the sample (**7**). A light intensity of 2.17 × 10^{16} photons s^{-1} cm^{-2} was employed for Φ_d determinations.

2.5. Theoretical calculations

Density functional theory (DFT) is one of the most useful quantum chemistry tools in calculating the ground state properties of compounds. In the modeling, the initial guess of the compound was provided from the X-ray coordinates. The molecular structures were optimized to get the global minima by using DFT/B3LYP/6-31G (d,p) level in the gas phase. The electronic properties were also calculated using 6-31G(d,p) and 6-31G+(d, p) levels. All the calculations were carried out with the Gaussian 16 B.01 [S6] package program and GaussView 6.0.16 [S7] was used for the visualization of the structure. The ^{1}H and ^{13}C NMR chemical shielding constants were calculated using GIAO-B3LYP in the gas phase and DMSO. For the NBO analysis, the same calculation procedure in the gas phase was also used. The ^{1}H and ^{13}C-NMR chemical shifts were converted to the TMS scale by subtracting the calculated absolute chemical shielding of TMS ($d = \Sigma_0 - \Sigma$), where d is the chemical shift, Σ is the absolute shielding and Σ_0 is the absolute shielding of TMS, whose values (reference shielding for ^{1}H and ^{13}C) are at 31.883 ppm and 191.80 ppm, respectively, for B3LYP/6-31G(d,p). Besides, molecular electrostatic potential (MEP) of the title molecules were investigated by B3LYP/6-31G(d,p). The energy difference between HOMO and LUMO levels was described as the optical bandgap for the HOMO to LUMO excitation energy (TDDFT) and the electronic band gap for excitation energy difference (ΔE = LUMO-HOMO). The visible absorption maxima of the molecule were corresponded to the electron transition from HOMO to LUMO by using calculations of molecular orbital geometry.

3. ^{1}H NMR, ^{13}C NMR, FT-IR, MS spectra and optimize geometric parameters of the ZnPc

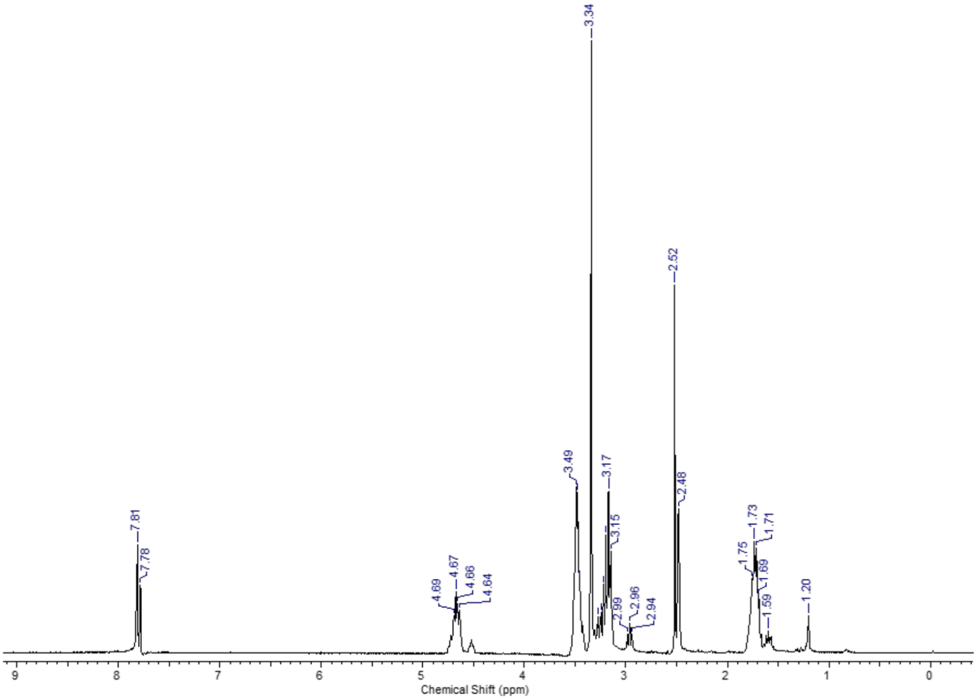

Figure S1. ^{1}H-NMR spectrum of 3,6-bis-(3-hydroxypropylthio)phthalonitrile (**3**) in DMSO-d_6.

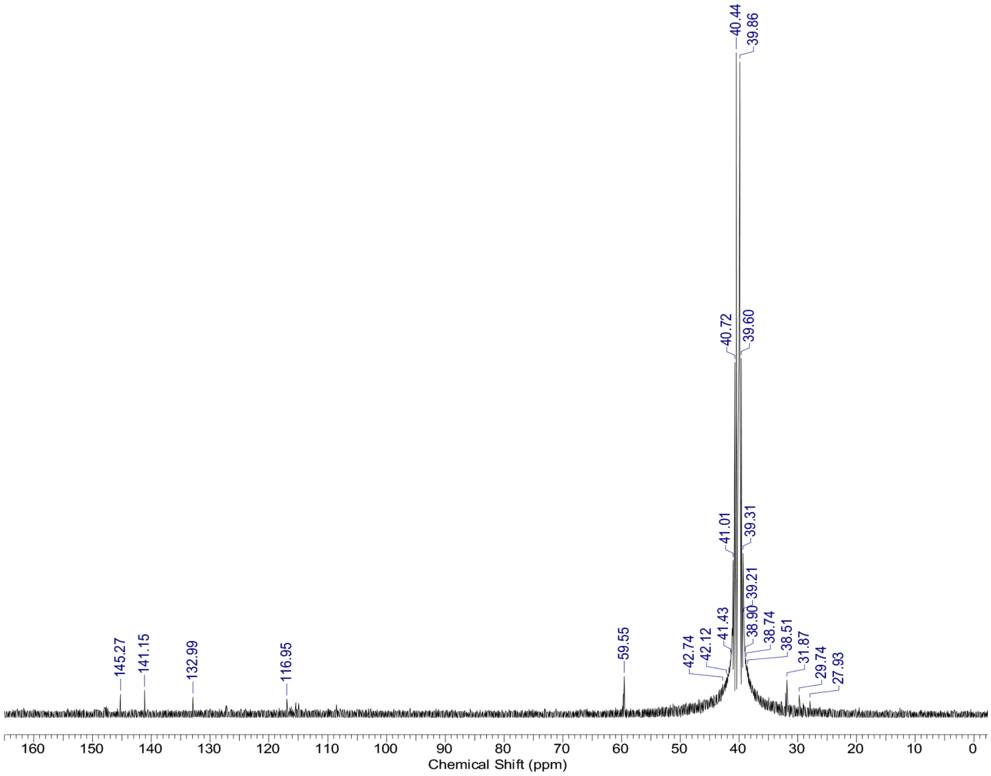

Figure S2. ^{13}C-NMR spectrum of 3,6-bis-(3-hydroxypropylthio)phthalonitrile (**3**) in DMSO-d_6.

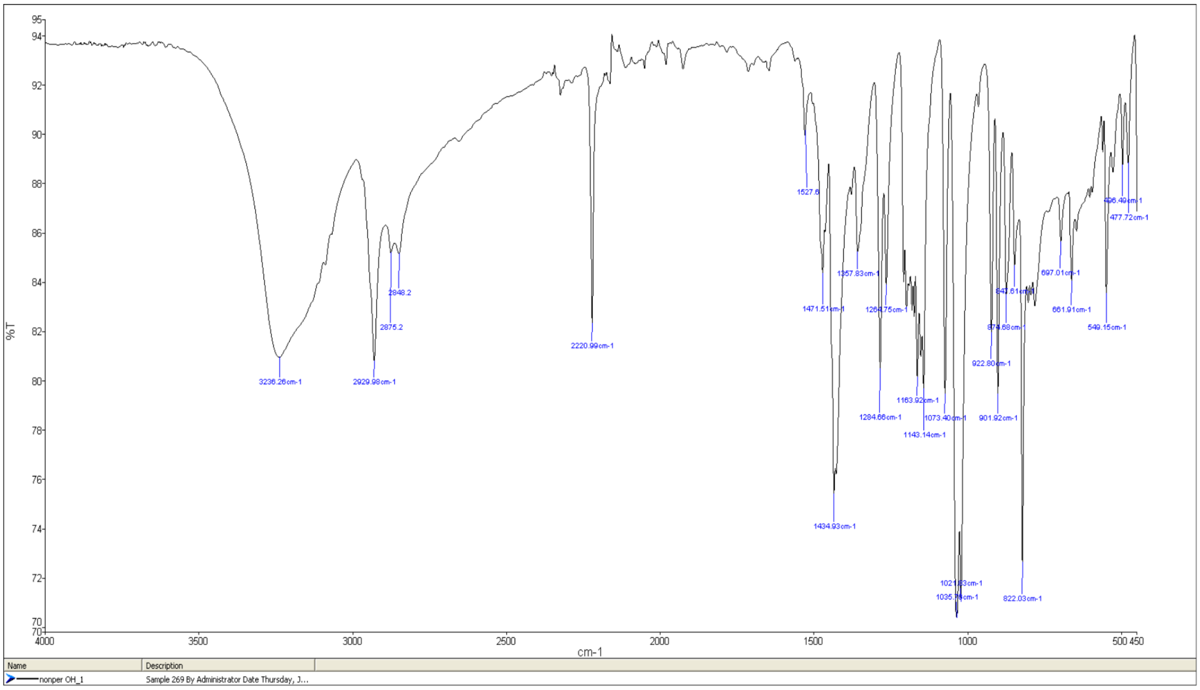

Figure S3. FT-IR spectrum of 3,6-bis-(3-hydroxypropylthio)phthalonitrile (**3**).

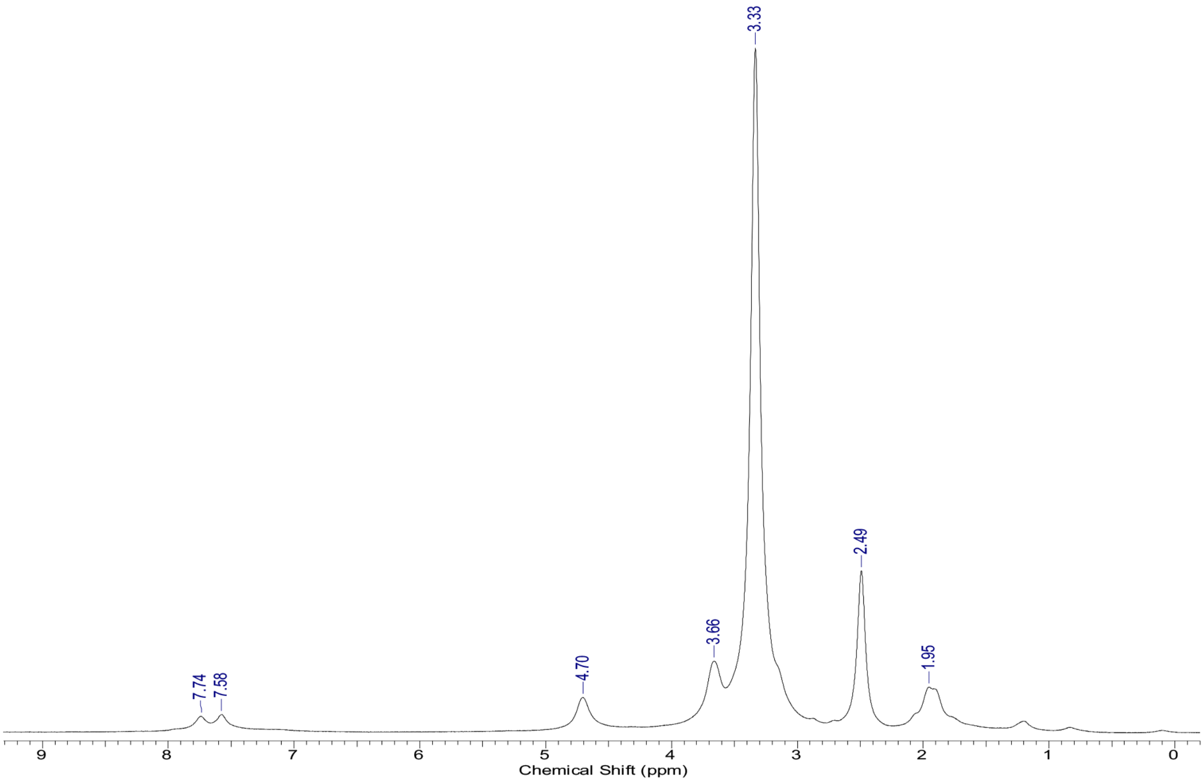

Figure S4. ^{1}H-NMR spectrum of **NiPc** in DMSO-d_6.

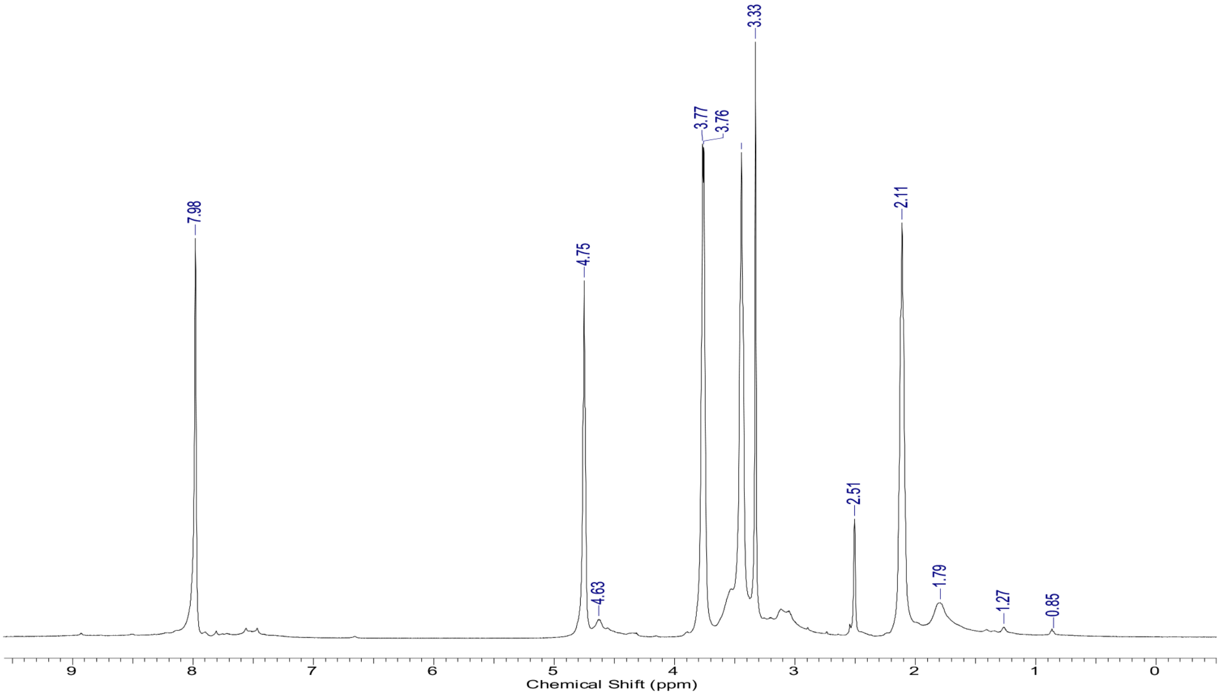

Figure S5. ^{1}H-NMR spectrum of **ZnPc** in DMSO-d_6.

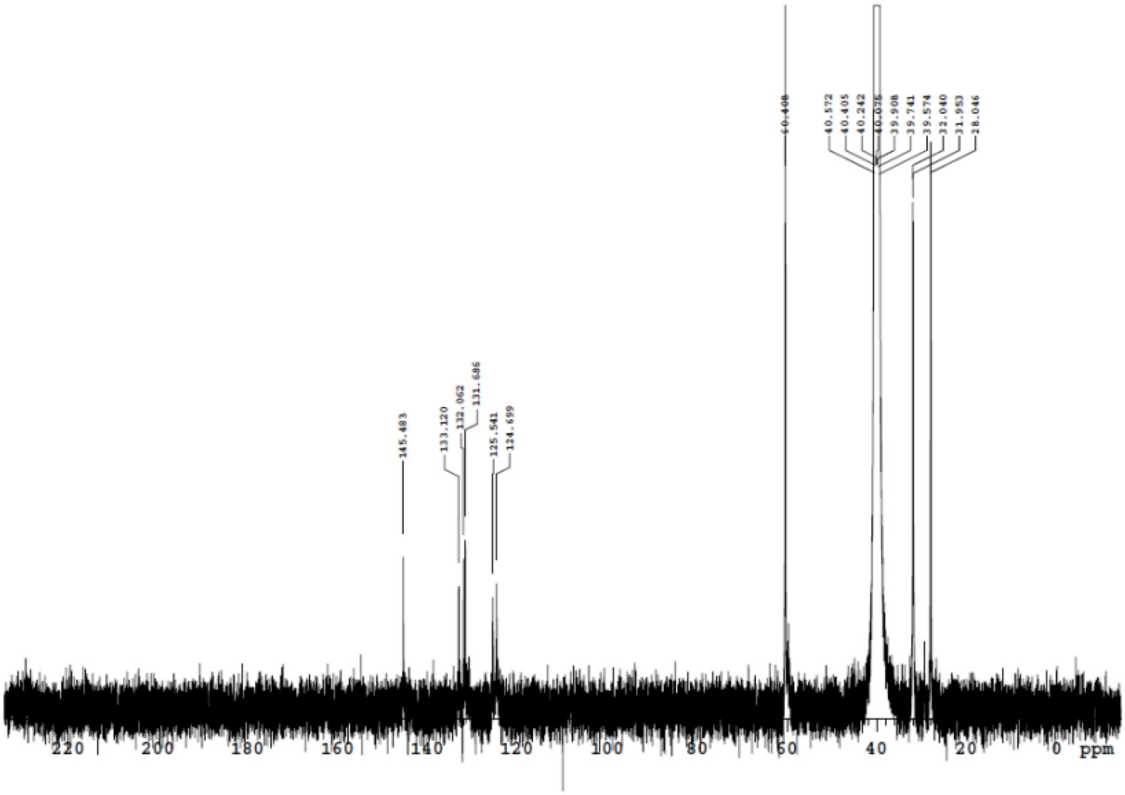

Figure S6. ^{13}C-NMR spectrum of **NiPc** in DMSO-d_6.

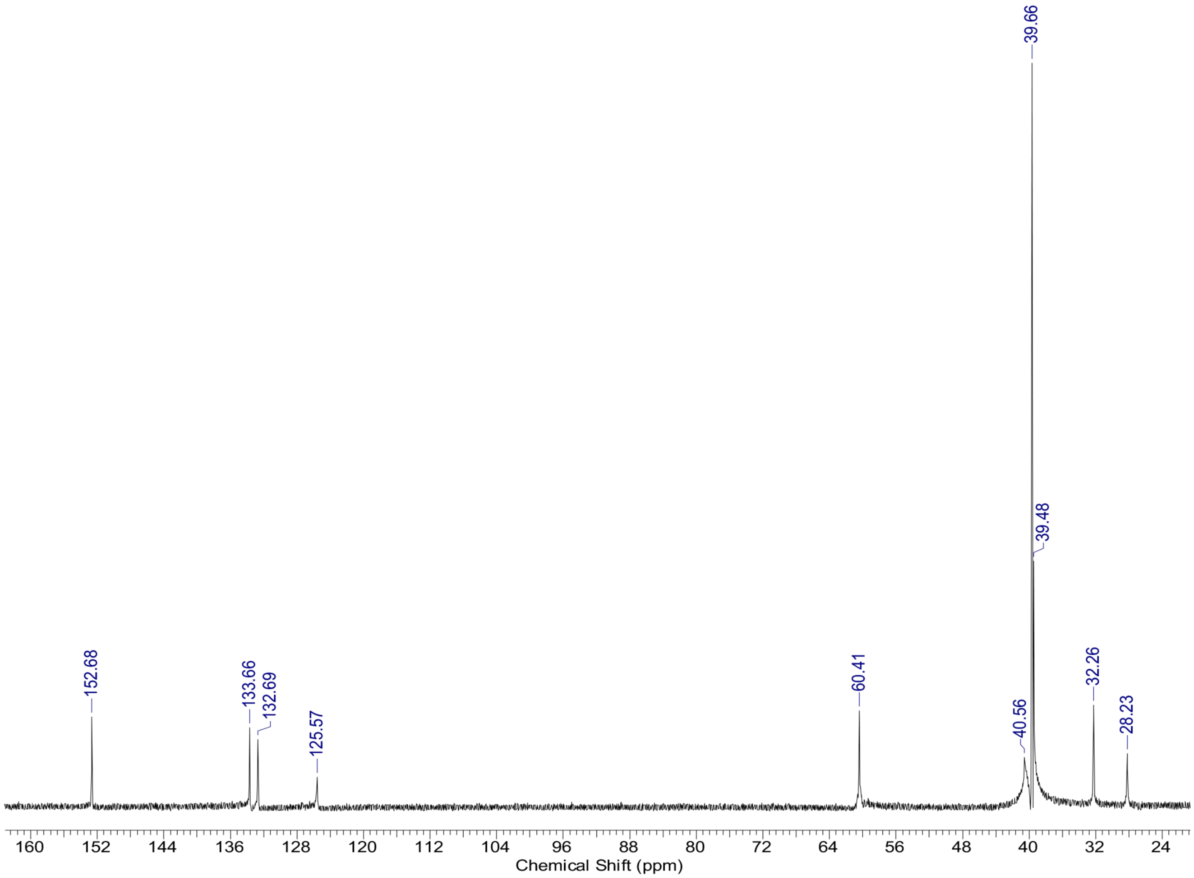

Figure S7. ^{13}C-NMR spectrum of **ZnPc** in DMSO-d_6.

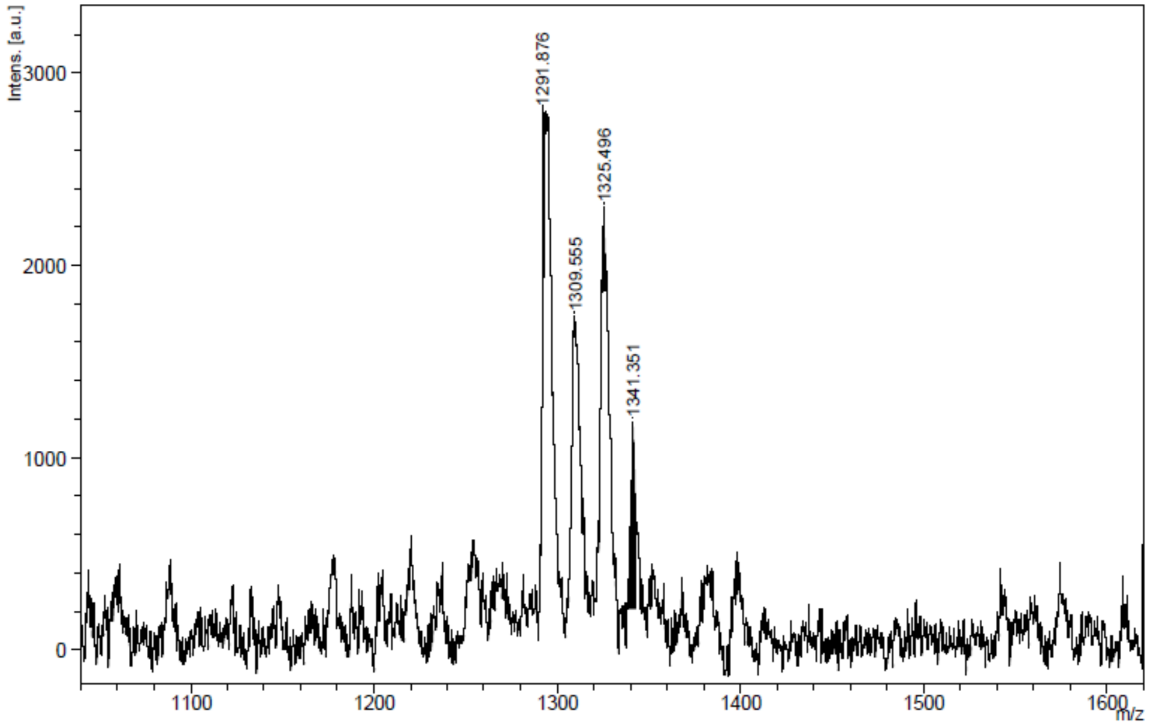

Figure S8. Mass spectrum of **NiPc** (MALDI-TOF).

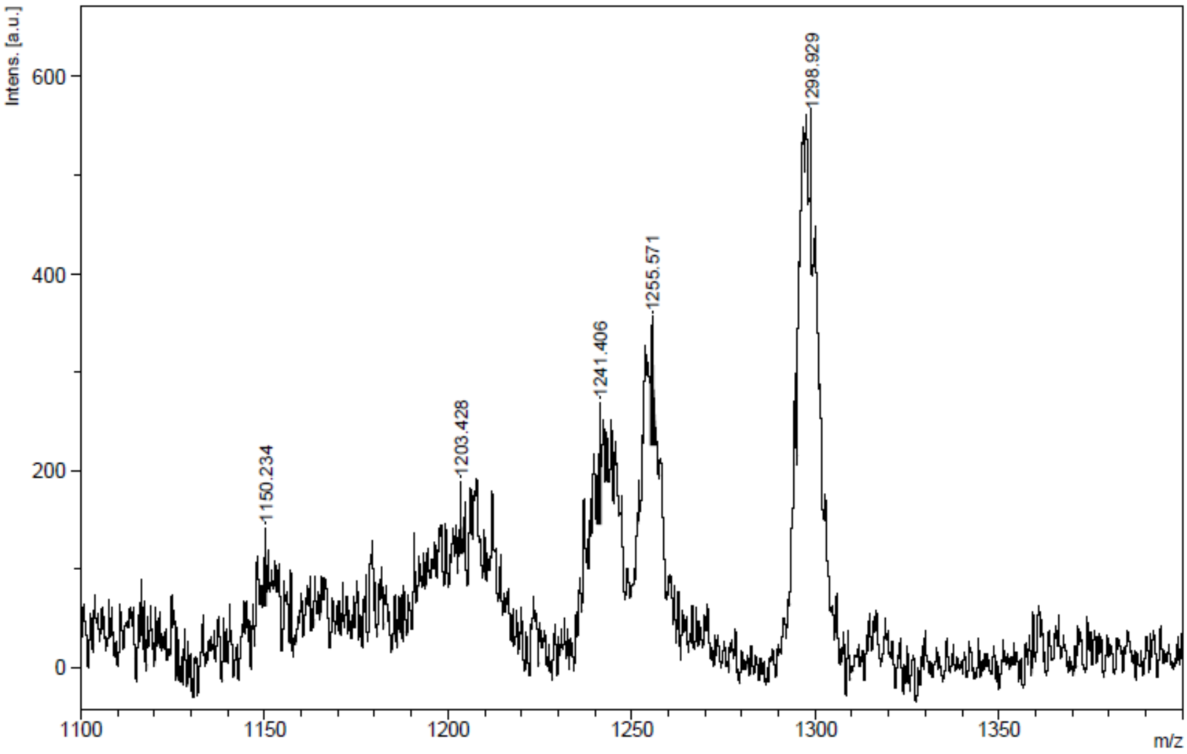

Figure S9. Mass spectrum of **ZnPc** (MALDI-TOF).

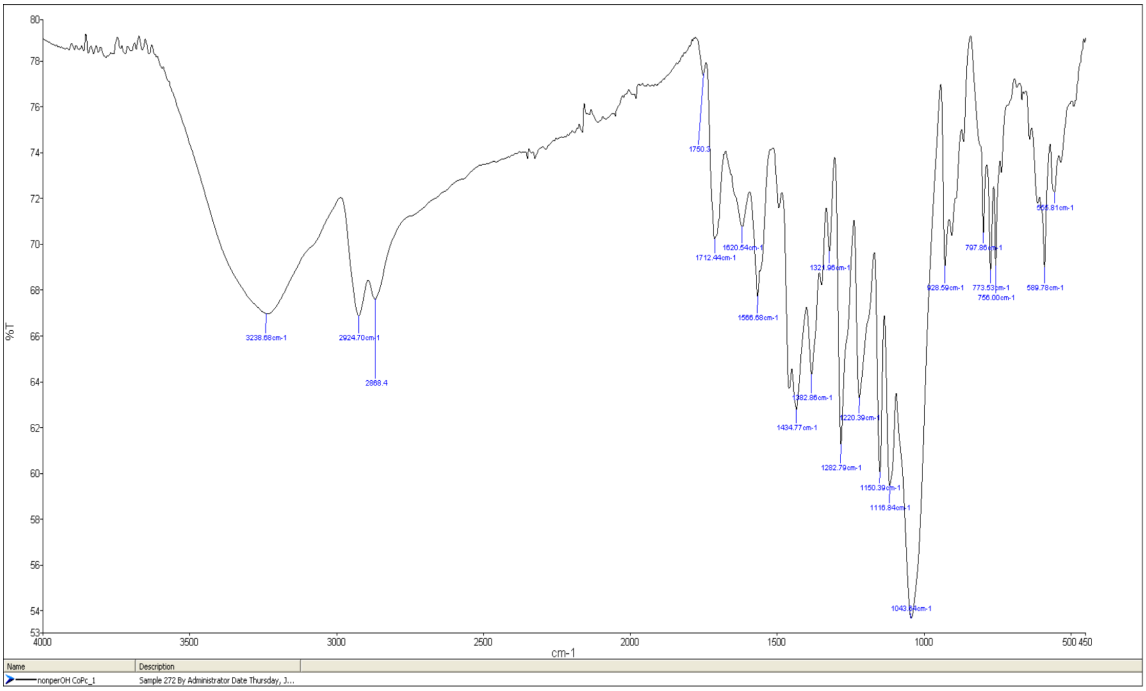

Figure S10. FT-IR spectrum of **CoPc**.

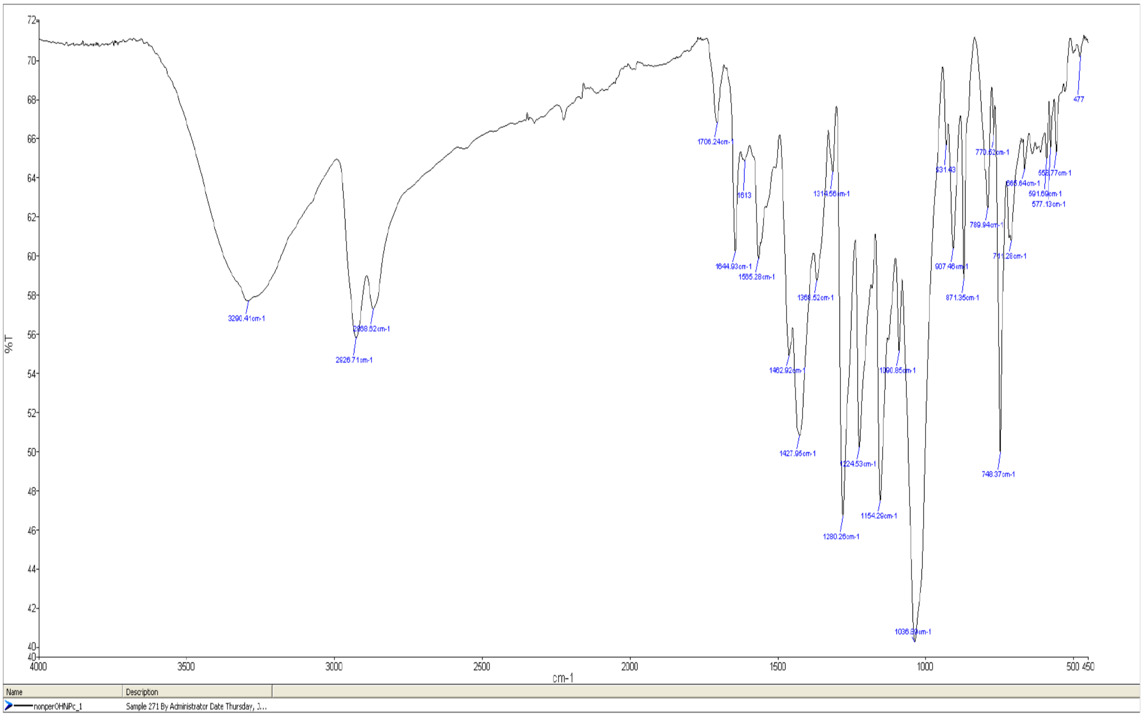

Figure S11. FT-IR spectrum of **CuPc**.

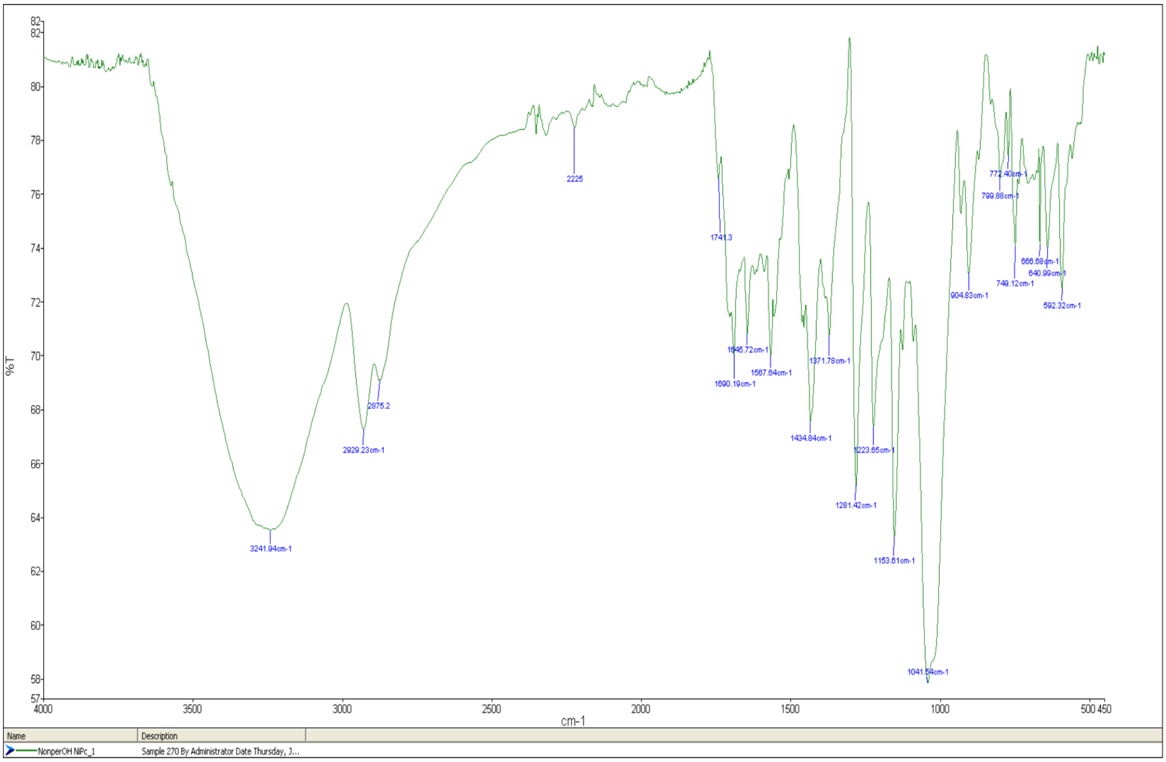

Figure S12. FT-IR spectrum of **NiPc**.

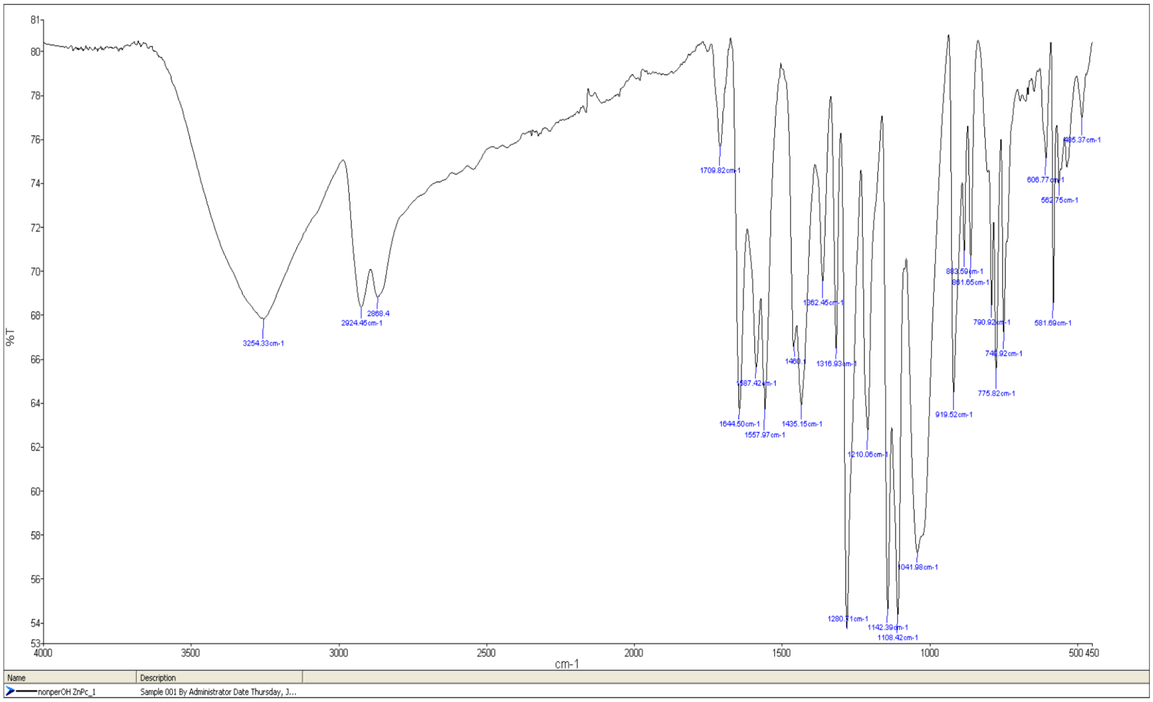

Figure S13. FT-IR spectrum of **ZnPc**.

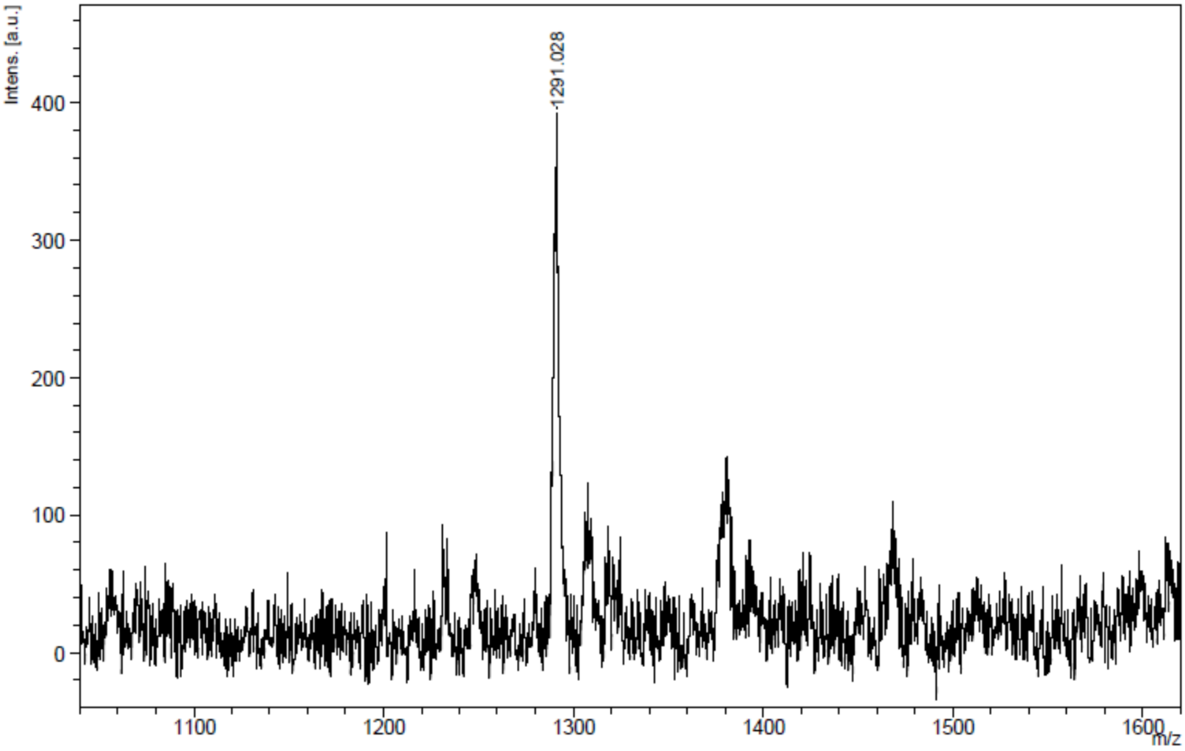

Figure S14. Mass spectrum of **CoPc** (MALDI-TOF).

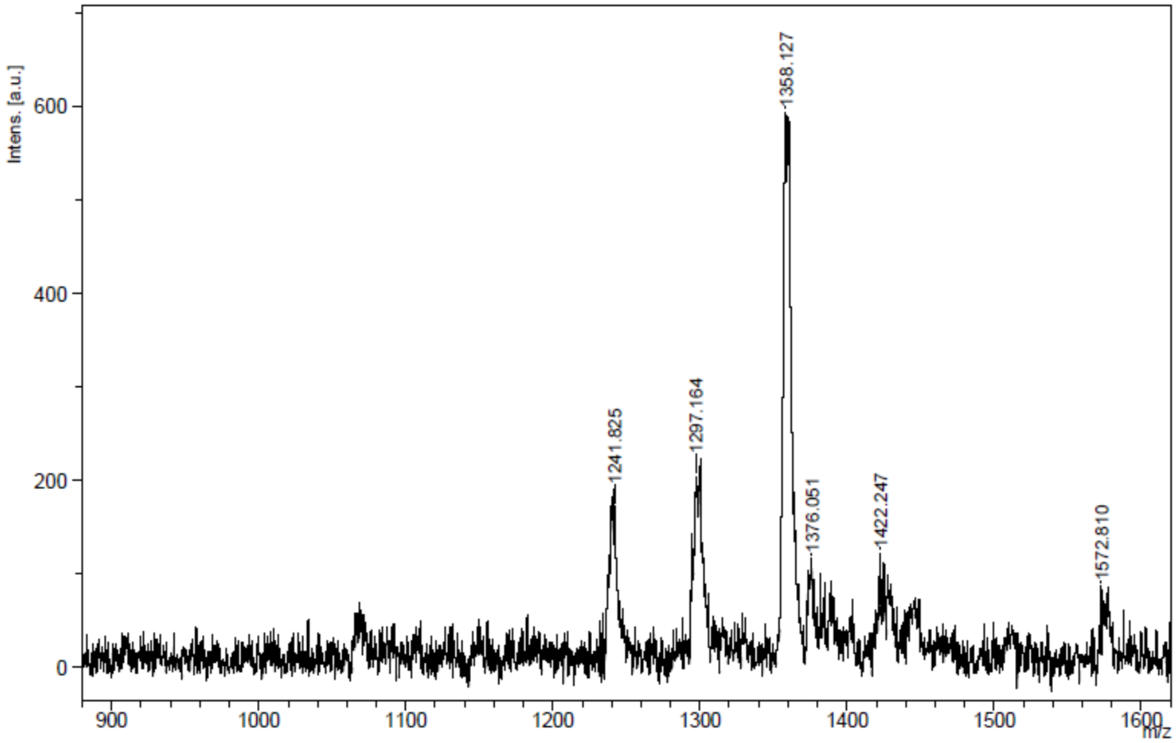

Figure S15. Mass spectrum of **CuPc** (MALDI-TOF).

Table S1. Selected optimized geometric parameters of the **ZnPc** in the ground state.

Bond lengths	(Å)	Bond Angles	(°)	Dihedral angles	(°)
C3-N5	1.33	C3-N7-Zn145	124.35	C14-N16-Zn145-N10	−173.72
N5-C20	1.33	C4-N7-Zn145	124.38	C19-N22-Zn145-N7	−173.71
C4-N6	1.33	C8-N10-Zn145	124.39	C8-N10-Zn145-N16	−171.47
C13-N15	1.33	C9-N10-Zn145	124.36	C4-N7-Zn145-N22	−171.45
C8-N21	1.33	C13-N16-Zn145	124.16	C2-C9-N10-Zn145	−162.30
N6-C14	1.33	C14-N16-Zn145	124.33	C2N3-N3-N7-Zn145	−162.25
C9-N15	1.33	C19-N22-Zn145	124.33	C11-C13-N16-Zn145	−160.86
C19-N21	1.33	C20-N22-Zn145	124.16	C18-C20-N22-Zn145	−160.84
C3-N7	1.37	N7-Zn145-N16	89.96	N21-C19-N22-Zn145	−22.14
C8-N10	1.37	N7-Zn145-N22	90.03	N6-C14-N16-Zn145	−22.14
C4-N7	1.37	N10-Zn145-N16	90.04	N6-C4-N7-Zn145	−20.43
C19-N22	1.37	N10-Zn145-N22	89.96	N21-C8-N10-Zn145	−20.40
C20-N22	1.37			C20-N22-Zn145-N7	−11.15
C13-N16	1.37			C13-N16-Zn145-N10	−11.14
C14-N16	1.37			N3-N7-Zn145-N22	−7.85
C9-N10	1.37			C9-N10-Zn145-N16	−7.82
N22-Zn145	1.99			C8-N10-Zn145-N22	8.44
N7-Zn145	1.99			C4-N7-Zn145-N16	8.46
N10-Zn145	1.99			C14-N16-Zn145-N7	10.41
N16-Zn145	1.99			C19-N22-Zn145-N10	10.42
				N15-C9-N10-Zn145	20.17
				N5-N3-N7-Zn145	20.22
				N15-C13-N16-Zn145	22.62
				N5-C20-N22-Zn145	22.65
				C17-C19-N22-Zn145	160.99
				C12-C14-N16-Zn145	161.01
				C24-C4-N7-Zn145	162.19
				C1-C8-N10-Zn145	162.23
				N3-N7- n145-N16	172.07
				C9-N10-Zn145-N22	172.10
				C20-N22-Zn145-N10	172.99
				C13-N16-Zn145-N7	172.99

Table S2. ^{1}H and ^{13}C chemical shift values of the **ZnPc** compound.

Atoms	Exp	Gas-phase	DMSO
C73, C74, C75, C72, C76, C77, C78, C79,	28.23	33.48	34.1
C57, C58, C61, C62, C63, C64, C59, C60	32.26	32.36	32.78
C97, C98, C99, C102, C103, C104	60,41	63.14	62.96
C1, C2, C11, C12, C17, C18, C23, C24	125.57	130.46	130.59
C25, C28, C29, C30, C31, C32, C33, C34	132.69	135.07	134.94
C26, C27, C35, C36, C37, C38, C39, C40	133.66	117.07	119.32
C3, C4, C13, C14, C8, C9, C19, C20	152.68	148.46	149.21
H81, H114, H82, H113, H83, H120, H96, H119, H95, H118, H94, H117, H84, H116, H80, H115	2.11	2.46	2.19
H122, H124, H126, H128, H130, H132, H134, H136	4.75	0.19	0.75
H66, H85, H86, H87, H65, H88, H89, H71, H70, H90, H69, H91, H68, H92, H67, H93	3.45	3.07	3.19
H107, H139, H106, H140, H109, H141, H105, H142, H110, H143, H111, H144, H108, H137, H112, H138	3.77	4.07	4.08
H41, H42, H43, H44, H45, H46, H47, H48	7.98	7.79	7.99

Table S3. Second order perturbation theory analysis of Fock matrix in NBO for the **ZnPc** in gas phase at B3LYP/6-31G(d,p).

Bond	Occupancy	Hybrid (p % ch.)	Acceptor (j)	Occupancy	Hybrid (p %ch.)	$E(^{2})^{a}$ (kcal/mol)	E(j)-E(i)[b] (a.u.)	F(i.j)[c] (a.u.)
C1-C2(π)	1.62025	p	π *(C9-N15)	0.45979	p	4.26	1.21	0.064
			σ *(C28-S49)	0.03155	$Sp^{2.66}$	4.05	0.87	0.053
			σ * (C8-N10)	0.04379	$Sp^{2.24}$	28.39	0.22	0.075
C8-N10 (σ *)	0.04379	$Sp^{2.24}$	π* (C1-C 2)	0.46887	p	68.90	0.05	0.073
			π* (C9-N15)	0.45979	p	91.22	0.03	0.060
			π*(C19-N21)	0.45985	p	190.70	0.03	0.087
C9-N15(π)	1.70502	p	π*(C13-N16)	0.58427	p	35.59	0.28	0.097
C20-N22(π)	1.76914	p	π*(C20-N22)	0.58427	p	32.10	0.32	0.096
C3-N5 (π*)	0.45979	p	π*(C23-C31)	0.46530	p	219.59	0.02	0.078
C 4-N7 (π*)	0.58474	p	π*(C3-N5)	0.45979	p	91.22	0.03	0.060
			π*(N6-C14)	0.45985	p	190.70	0.03	0.087
			π*(C24-C32)	0.46530	p	99.63	0.04	0.080
C9-N15(π*)	0.45979	p	π*(C1-C2)	0.46887	p	118.85	0.02	0.070
C13-N16 (π*)	0.58427	p	π*(N6-C 14)	0.45985	p	91.52	0.03	0.060
			π*(C9-N15)	0.45979	p	191.05	0.03	0.087
			π*(C11-C34)	0.46132	p	97.65	0.04	0.080
C19-N21 (π*)	0.45985	p	π*(C17-C29)	0.46132	p	209.25	0.02	0.078
C20-N22(π*)	0.58427	p	π*(C3-N5)	0.45979	p	191.04	0.03	0.087
			π*(C18-C30)	0.46132	p	97.65	0.04	0.080
			π*(C19-N21)	0.45985	p	91.52	0.03	0.060
C25-C26 (π*)	0.40404	p	π*(C1-C2)	0.46887	p	275.24	0.01	0.079
C27-C28 (π*)	0.40404	p	π*(C1-C2)	0.46887	p	275.24	0.01	0.079
N6 (LP1)	1.86832	$Sp^{3.13}$	σ*(C4-N7)	0.04379	$Sp^{2.24}$	14.48	0.80	0.098
			σ*(C14-N16)	0.04373	$Sp^{2.25}$	14.55	0.80	0.099
S 49 (LP2)	1.82989	p	π*(C 27-C28)	0.40404	p	20.52	0.25	0.068
S 56 (LP2)	1.83354	p	π* (C12-C33)	0.46132	p	18.28	0.25	0.066

$E^{(2)a}$ means energy of hyperconjugative interaction; [b]energy difference between donor and acceptor i and j NBO orbital; F(i,j)[c] is the Fock matrix element between i and j NBO orbitals.

References

S1. Perrin DD, Armarego WLF. Purification of Laboratory Chemicals. 2nd ed. Oxford, UK: Pergamon Press, 1980.

S2. Wang R, Zhao Y, Zhu C, Huang X. New synthesis of 3,6-dibromophthalonitrile and phthalocyanine having eight thienyl substituents at peripheral α-positions. Journal of Heterocyclic Chemistry 2015; 52 (4): 1230-1233. doi: 10.1002/jhet.2130

S3. Baygu Y, Gök Y. A highly water-soluble zinc(II) phthalocyanines as potential for PDT studies: synthesis and characterization. Inorganic Chemistry Communication 2018; 96: 133-138. doi: 10.1016/j.inoche.2018.08.004

S4. Durmuş M. Photochemical and Photophysical Charactrization. In: Nyokong T, Ahsen V (editors). Photosensitizers in Medicine, Environment and Security. New York, NY, USA: Springer, 2012, pp. 135-266.

S5. Spiller W, Kliesch H, Wöhrle D, Hackbarth S, Schnurpfeil G. Singlet oxygen quantum yields of different photosensitizers in polar solvents and micellar solutions. Journal of Porphyrins and Phthalocyanines 1998; 2 (2): 145-158. doi: 10.1002/(SICI)1099-1409(199803/04)2:2<145::AID-JPP60>3.0.CO;2-2

S6. Frisch MJ, Trucks GW, Schlegel HB, Scuseria GE, Robb MA et al. Gaussian 16, revision B.01. Wallingford, CT, USA: Gaussian Inc., 2016.

S7. Dennington RD, Keith TA, Millam JM. GaussView 6.0.16. Shawnee, KS, USA: Semichem, Inc., 2000-2016.
